# Neurocognitive and Behavioral Outcomes of Chinese Survivors of Childhood Lymphoblastic Leukemia

**DOI:** 10.3389/fonc.2021.655669

**Published:** 2021-04-20

**Authors:** Liwen Peng, Lok Sum Yang, Perri Yam, Chun Sing Lam, Agnes Sui-yin Chan, Chi Kong Li, Yin Ting Cheung

**Affiliations:** ^1^ School of Pharmacy, Faculty of Medicine, The Chinese University of Hong Kong, Hong Kong, China; ^2^ Neuropsychology Laboratory, Department of Psychology, The Chinese University of Hong Kong, Hong Kong, China; ^3^ Chanwuyi Research Center for Neuropsychological Well-Being, The Chinese University of Hong Kong, Hong Kong, China; ^4^ Department of Pediatrics, Faculty of Medicine, The Chinese University of Hong Kong, Hong Kong, China; ^5^ Hong Kong Hub of Paediatric Excellence, The Chinese University of Hong Kong, Hong Kong, China; ^6^ Department of Paediatrics and Adolescent Medicine, The Hong Kong Children’s Hospital, Hong Kong, China

**Keywords:** cognitive, function, behavior, childhood cancer, childhood acute lymphoblastic leukemia (ALL), survivorship

## Abstract

**Background:**

Increasing attention has been dedicated to investigate modifiable risk factors of late effects in survivors of childhood cancer. This study aims to evaluate neurocognitive and behavioral functioning in a relatively young cohort of survivors of childhood acute lymphoblastic leukemia (ALL) in Hong Kong, and to identify clinical and socio-environmental factors associated with these outcomes.

**Methods:**

This analysis included 152 survivors of childhood ALL who were ≥5 years post-diagnosis (52% male, mean [SD] age 23.5[7.2] years at evaluation, 17.2[7.6] years post-diagnosis). Survivors completed performance-based neurocognitive tests, and reported their emotional and behavioral symptoms using the Child/Adult Behavior Checklist. Socio-environmental variables (living space, fatigue, physical activity, family functioning, and academic stress) were self-reported using validated questionnaires. Clinical variables and chronic health conditions were extracted from medical charts. Multivariable linear modeling was conducted to test identify factors associated with neurocognitive/behavioral outcomes, adjusting for current age, sex, age at diagnosis and cranial radiation. An exploratory mediation analysis was performed to examine the mediating effects of risk factors on neurocognitive and behavioral outcomes.

**Results:**

As compared to population norms, a minority of survivors developed mild-moderate impairment in motor processing speed (36.2%), memory (9.2%) and attention measures (4.0%-10.5%). Survivors also reported attention problems (12.5%), sluggish cognitive tempo (23.7%) and internalizing (depressive, anxiety and somatic symptoms) problems (17.1%). A minority of survivors developed mild-moderate treatment-related chronic conditions (n=37, 24.3%). As compared to survivors without chronic conditions, survivors with chronic conditions had more executive dysfunction (B=5.09, standard error [SE]=2.05; *P*=0.014) and reported more attention problems (B=5.73, SE=1.43; *P*<0.0001). Fatigue and poor family functioning was associated with multiple measures of behavior problems (all *P*<0.001). A lower level of physical activity was correlated with more self-reported symptoms of inattention (B= -1.12, SE=0.38, *P*=0.004) and sluggish cognitive tempo (B=-1.22, SE=0.41, *P*=0.003). Exploratory analysis showed that chronic health conditions were associated with behavioral measures through fatigue as the mediator.

**Conclusion:**

The majority of young Chinese survivors of ALL had normal cognitive and behavioral function. Regular monitoring of behavioral function should be performed on survivors who develop treatment-related chronic conditions. Health behavior and socio-environment factors may be potentially modifiable risk factors associated with health outcomes in survivors.

## Introduction

Improved treatment strategies for childhood acute lymphoblastic leukemia (ALL) have yielded survival rates higher than 90% (1). However, survivorship may be complicated by a myriad of treatment-related adverse effects (2, 3). Most current survivors of childhood ALL receive contemporary treatments that eliminate the need for cranial radiation therapy (CRT). Still, long-term survivors of childhood ALL who have been treated with contemporary chemotherapy protocols exhibit mild to moderate neurocognitive impairment (4, 5). The rates of neurocognitive deficits affecting executive function, processing speed and memory are threefold higher among survivors of childhood ALL than the general population (4, 6–8). Survivors also demonstrate behavioral and psychological problems (9–11), as well as worse academic performance (12, 13).

The association of central nervous system (CNS)-directed therapies, such as high-dose methotrexate and intrathecal chemotherapy, with worse neurocognitive outcomes in survivors of childhood ALL is well established (4, 6–8, 14–16). Leukoencephalopathy, sepsis and other acute toxicities that occur during active treatment are predictive of structural changes in the brain and subsequent deficits in functional outcomes (9, 17–19). After treatment, aging survivors of childhood cancer develop chronic health conditions, such as cardiovascular, pulmonary and metabolic disorders, at higher rates than those observed in age-matched non-cancer siblings (20, 21). In addition to their associations with early mortality, emerging studies of survivors have shown that these chronic health problems are related to cognitive impairment and psychosocial difficulties (15, 22–25).

To date, the majority of cognitive studies have involved Western populations. However, a recent systematic review identified 13 cognitive studies in survivors of childhood cancer in Asian countries, and found that 10.0%–42.8% of survivors demonstrated mild-to-moderate impairments in intelligence (i.e., overall IQ) (26). Evidence obtained in a Western population cannot be extrapolated to Asian survivors because of genetic differences in responses to drug therapies and susceptibilities to developing treatment-related chronic toxicities (27). Ethnic and sociocultural factors may lead to differential effects of treatments on cognitive processes in Asian and Western survivors (28, 29). Cultural values and family relationships may also shape psychosocial development (30). However, few studies have systematically evaluated the multifactorial aspects of cognitive and psychosocial outcomes in Asian survivors of childhood cancer.

Notably, most research has focused on either disease- or treatment-related factors as predictors of cognitive dysfunction. Few studies have examined the mediating effects of socio-environmental factors on the functional outcomes of survivors (31). This is especially relevant in the Asian context, in which great emphasis has been placed on ameliorating the adverse health effects of an urban environment, such as sleep disturbances, a sedentary lifestyle and academic stress, on children and adolescents (26, 32). Particularly, poor environmental factors and health behaviors may influence poorer neurocognitive and behavioral functions, especially in survivors who are already at risk of adverse health outcomes due to the cancer and related treatment.

In Hong Kong, approximately 50 pediatric patients are diagnosed with leukemia each year (33). The survival rate of patients with childhood ALL in Hong Kong is comparable to those in other developed countries, and more than 90% of patients survive more than 5 years after diagnosis (34). Currently, no studies have systematically characterized the functional outcomes in this population. The objectives of this study were to evaluate the prevalence of neurocognitive and behavioral deficits, and to identify clinical and socio-environmental factors associated with these outcomes in a cohort of young Chinese survivors of childhood ALL. We also included an exploratory objective to examine the mediating effects of socio-environmental factors on neurocognitive and behavioral outcomes in this population.

## Methods

### Study Design

This was a prospective, cross-sectional study conducted at the Long-term Follow-up (LTFU) Clinic of the Prince of Wales Hospital in Hong Kong. This regional tertiary care public hospital serves as a major hub providing LTFU care to survivors of childhood cancer. This study was approved by the Joint Chinese University of Hong Kong – New Territories East Cluster Clinical Research Ethics Committee. Written informed consent and assent were obtained from all adult and pediatric participants, respectively.

### Study Population

Eligible participants were recruited through consecutive sampling. Between June 2019 and June 2020, investigators obtained the list of patients who were due for follow-up consultation at the LTFU clinic, which typically occurred once a week. Patients were then screened for eligibility using the in-house electronic patient record system (Clinical Management System [CMS]). All eligible patients who subsequently attended the LTFU clinic were invited to participate in the study.

Survivors were eligible for the study if they (1) were at least 12 years old during the time of recruitment, (2) had been diagnosed with ALL before the age of 18 years, and (3) had survived for at least 5 years since diagnosis or had completed treatment at least 2 years previously. We excluded survivors who (1) had relapsed, (2) developed secondary malignancies, (3) had any genetic disorder or pre-existing condition associated with cognitive impairment (e.g., Down syndrome), (4) were pregnant or lactating or (5) had a history of traumatic brain injury.

Of the 192 survivors who were screened for eligibility, 175 fulfilled the inclusion criteria and were invited to participate in the study (**Supplement 2**). Subsequently, 158 survivors completed the assessments. After excluding 6 of those survivors because of missing diagnosis or treatment protocol information, the data of 152 survivors were analyzed (response rate: 86.9%). The study cohort (**Table 1**) comprised 32 pediatric survivors (mean age = 14.0, SD = 2.2 years) and 120 adult survivors (mean age = 26.0, SD = 5.9 years). On average, they were 17.2 (SD = 7.6) years post-cancer diagnosis, and 15.3 (SD = 11.2) years had elapsed since treatment (**Table 1**).

**Table 1:** Clinical and Treatment Characteristics of Study Cohort (n=152).

Characteristics	No. (%)	Mean (SD)
**Demographics and Clinical**		
**Sex**		
**Male**	79 (52.0)	–
**Female**	73 (48.0)	–
**Highest education (years)**	–	13.2 (3.3)
**Age at diagnosis (years)**	–	6.3 (4.3)
**Age at evaluation (years)**	–	23.5 (7.2)
**≤ 18**	32 (21.0)	–
**>18 – ≤30**	93 (61.2)	–
**>30**	27 (17.8)	–
**Time since diagnosis (years)**	–	17.2 (7.6)
**5 – ≤10**	27 (17.8)	–
**>10 – ≤15**	35 (23.0)	–
**>15 – ≤20**	44 (28.9)	–
**>20**	46 (30.3)	–
**Time since completion of treatment (years)**	–	15.3 (11.2)
**2 – ≤10**	39 (25.6)	–
**>10 – ≤15**	35 (23.0)	–
**>15 – ≤20**	43 (28.3)	–
**>20**	35 (23.0)	–
**Risk group**		
**Standard risk**	61 (40.1)	–
**Intermediate risk**	67 (44.0)	–
**High risk**	22 (14.1)	–
***Missing***	2 (1.3)	–
**Treatment modality**		
**Cranial radiation**	32 (21.0)	–
**Chemotherapy-only protocol**	120 (79.0)	–
**HSCT**	4 (2.6)	–
**Chemotherapy**		
IV daunorubicin/doxorubicin* (mg/m^2^)	–	194.4 (53.0)
IV high-dose methotrexate* (g/m^2^)	–	14.2 (6.3)
**8g/m^2^**	56 (36.9)	–
** 20 g/m^2^**	96 (63.1)	–
Intrathecal chemotherapy* (no. of injections)	–	17.6 (4.1)
**Chronic health conditions^**		
**Any**	37 (24.3)	–
**Cardiopulmonary**	13 (8.6)	–
** Grade 1/2 (mild-moderate)**	13 (8.6)	–
** Grade 3/4 (severe-life threatening)**	0	–
**Endocrine**	7 (4.6)	–
** Grade 1/2 (mild-moderate)**	7 (4.6)	–
** Grade 3/4 (severe-life threatening)**	0	–
**Metabolic**	7 (4.6)	–
** Grade 1/2 (mild-moderate)**	5 (3.3)	–
** Grade 3/4 (severe-life threatening)**	2 (1.3)	–
**Neurology**	10 (6.6)	–
** Grade 1/2 (mild-moderate)**	9 (5.9)	–
** Grade 3/4 (severe-life threatening)**	1 (0.6)	–
**Psychiatry**	9 (5.9)	–
** Grade 1/2 (mild-moderate)**	0	–
** Grade 3/4 (severe-life threatening)**	9 (5.9)	–
**Vision & Hearing**	3 (2.0)	–
** Grade 1/2 (mild-moderate)**	3 (2.0)	–
** Grade 3/4 (severe-life threatening)**	0	–

HSCT, hematopoietic stem cell transplantation; IV, intravenous; SD, standard deviation.

*Cumulative doses of selected chemotherapy drugs were extracted from medical charts, which were only available for 138 survivors. Cumulative doses for the remaining survivors (n=14) were estimated based on the chemotherapy protocol they received. Total HDMTX was also categorized as 8 g/m^2^ (4 cycles of 2 g/m^2^) of methotrexate versus 20 g/m^2^ (4 cycles of 5 g/m^2^).

^Conditions were graded for severity according to the National Cancer Institute’s Common Terminology Criteria for Adverse Events (CTCAE version 4.03).

### Previous Treatment Exposures

The treatment characteristics of the study cohort is presented in **Table 1**. All survivors in Hong Kong were treated with childhood ALL protocols (34) that were similar to the strategies adopted by international pediatric oncology organizations. Based on the clinical presentations of leukemia at diagnosis and evaluation of minimal residual disease, the patients were stratified into standard-risk, intermediate-risk, or high-risk protocols. Protocols were divided into four major components: remission induction block, consolidation block, maintenance block, and CNS-directed treatment. A variety of cytotoxic drugs were administered, which typically included intravenous (IV) high-dose methotrexate (HDMTX, defined as a single-dose of more than 1 g/m^2^ of methotrexate) with leucovorin rescue, intrathecal chemotherapy injections (either methotrexate, or a combination of methotrexate, hydrocortisone, and cytarabine), oral dexamethasone pulses, anthracyclines, l-asparaginase, cytarabine and cyclophosphamide. Children who were stratified into the intermediate-risk/high-risk protocols received longer duration of treatment with higher intensity of HDMTX (4 cycles of 5g/m^2^ HDMTX in intermediate-risk/high-risk protocols vs 4 cycles of 2g/m^2^ HDMTX in low-risk protocols), intrathecal chemotherapy and dexamethasone, in addition to other chemotherapeutic agents. A minority of children in the intermediate-risk/high-risk arm, especially those who were treated before 1995, received prophylactic CRT.

### Study Outcomes

Neurocognitive function was assessed using a standardized performance-based neurocognitive battery, which included: (1) *measures of attention* (Continuous Performance Test-III [CPT-III] variables: detectability, omissions, variability, hit reaction time block change and hit reaction time inter-stimulus interval) (35), (2) *memory* (Modified Taylor Complex Figure) (36), (3) *processing speed* (Trail Making Test Part A [TMT-A] and Grooved Pegboard) (37) and (4) *executive function* (Trail Making Test Part B [TMT-B] and CPT-III variables: commissions and preservations) (37). Detailed descriptions of the study tools and sources of reference norms data are reported in Supplement 1.

Behavioral functioning was evaluated using the Child Behavior Checklist (CBCL) and Adult Behavior Checklist (ABCL) for pediatric survivors (12 – 18 years of age) and adult survivor (≥ 18 years of age), respectively (38). The syndrome scales of the CBCL and ABCL were set as the primary behavioral outcomes of interest in this study. The standardized scales included attention problems, thought problems, internalizing problems (consisting anxiety/depression, somatic complaints and withdrawn behavior), externalizing problems (consisting aggressive behavior, intrusive behavior and rule-breaking behavior), obsessive-compulsive problems and sluggish cognitive tempo. Traditional Chinese-language versions of the ABCL and CBCL are available, and age- and gender-standardized local norms have been established for the Hong Kong context (39). The descriptions of ABCL/CBCL domains are presented in **Supplement 1**.

All neurocognitive and behavioral measures were transformed into age-adjusted *T*-scores (mean = 50; standard deviation [SD] = 10) using references provided by the test manuals or the published literature (**Supplement 1**). All *T*-scores were scaled such that a higher score was indicative of worse cognitive functioning or more severe problems. To estimate the prevalence of impairments within the study sample, impairment was defined as a score worse than 1.5 standard deviations of the age-adjusted *T*-score, a definition that has been adopted by multiple studies involving childhood cancer survivors in the literature (6, 10, 15, 18).

### Clinical and Treatment Variables

Demographic and cancer-related information was abstracted from the Clinical Management System (CMS), an electronic health data repository of the public healthcare system in Hong Kong. This database is considered a reliable data source for epidemiological research in Hong Kong (40). The CMS includes cancer-related variables (diagnoses, age at diagnosis, risk stratification) and treatment-related variables (chemotherapy drugs, cumulative doses, CRT).

Information about chronic health conditions was collected from the CMS and through patient/proxy interviews. The severity of the conditions was graded according to the National Cancer Institute’s Common Terminology Criteria for Adverse Events (CTCAE version 4.03) (41), which defines the severity of the health conditions as asymptomatic/mild symptoms (grade 1), moderate symptoms requiring minimal interventions (grade 2), severe/disabling symptoms requiring extensive interventions (grade 3), and life-threatening conditions (grade 4). For this study, the health conditions of interest were limited to the cardiopulmonary, endocrine, metabolic, psychiatric, neurological and hearing/vision systems, as these have been associated with neurocognitive function in cancer survivors (15, 22, 23, 25, 42). Only chronic health conditions with a reported age of onset during or after the completion of cancer treatment were included.

### Socio-Environmental Variables

Family functioning was assessed using the Chinese Family Assessment Instrument (CFAI) (43). The CFAI is a 33-item tool that measures the domains of mutuality, communication and cohesiveness, conflict and harmony, parental concern and parental control. It has been validated within the general population in Hong Kong, with satisfactory test–retest reliability (Cronbach’s alpha = 0.96) (44). The item scores are summed to yield total scores ranging from 33 to 165, and a higher score represents poorer family functioning.

Physical activity was self-reported using the validated Chinese University of Hong Kong: Physical Activity Rating for Children and Youth (CUHK-PARCY) (45, 46), which uses an 10-point scale to evaluate the level, intensity and frequency of physical activity performed by children and adolescents. The scale ranges from 0 to 10, with a higher rating indicating a more physically active lifestyle.

The subjective fatigue level was assessed using the Pediatric Quality of Life Inventory Multidimensional Fatigue Scale (PedsQL MFS) (47, 48). This scale comprises 18 items that evaluate the subjective sleep-rest fatigue, general fatigue and cognitive fatigue. The PedsQL MFS has demonstrated good internal consistency reliability, content validity and construct validity in the Chinese pediatric and young adult population (49). Each item is scored on a 100-point reverse Likert scale. A lower score indicates more severe fatigue.

Academic stress in survivors who were still attending school was assessed using the Education Stress Scale for Adolescents (ESSA). The 16-item ESSA addresses five latent variables: pressure related to studies, academic workload, concern about grades, self-expectation and despondency (50). The ESSA has been culturally adapted and validated in Chinese adolescents. The scale showed good internal consistency (Cronbach’s alpha = 0.81) and test–retest reliability (intraclass correlation coefficient = 0.78) in Chinese adolescents (50). The total score ranged from 16 to 80, with a higher score indicating greater academic stress.

### Sample Size

The primary outcome of interest was a statistical difference in the mean cognitive scores between survivors and reference norms (mean = 50, SD = 10). At the inception of this study, there were no studies that reported performance-based cognitive outcomes in Asian survivors of childhood ALL. Hence, sample size calculation was based Western survivors of childhood ALL who received similar treatment regimens and neurocognitive testing at follow-up (7). The mean differences in cognitive scores between survivors and norms ranged from 0.25 to 2.3 SDs. Conservatively, to detect a 0.25 SD difference in scores between survivors and norms with a significance level α=0.05, the required sample size was 126 survivors.

### Statistical Analysis

The sample characteristics and outcomes measures are summarized using descriptive statistics. As CRT is a well-established predictor of brain outcomes in survivors of childhood cancer (7, 15, 22), the clinical characteristics and neurocognitive/behavioral outcomes are presented separately for survivors who did and did not undergo CRT (CRT and non-CRT groups, respectively). The Mann-Whitney U test and Chisquare test were used to compare differences in characteristics between CRT and non-CRT groups.

A one-sample *t* test was used to compare the survivors’ performance with population norms (*T*-score = 50). Only measures on which the survivors differed from the normative samples after correcting for false discovery rate (51), and demonstrated an impairment rate of more than 5% were included in subsequent analyses.

Multivariable general linear modeling (GLM) was used to identify the factors associated with neurocognitive and behavioral outcomes. The basic model included the current age, sex, age at diagnosis and CRT status. Subsequently, other independent variables of interest were added to the basic model and analyzed separately to avoid multi-collinearity. The following risk factors were determined *a priori* based on a literature review (6, 7, 10, 13, 16, 18, 22, 23, 52–54): (1) chronic health conditions; (2) treatment factors, including risk stratification, the IV HDMTX dose (8 g/m^2^ vs 20 g/m^2^) and number of intrathecal chemotherapy injections; and (3) socio-environmental factors, including fatigue, living space (≤ 600 vs > 600 square feet [55 m^2^]), family functioning, physical activity and academic stress (only for current students). Unstandardized point estimates (B) and standard errors (SE) were used to quantify the effect size of the associations. Correction was not conducted for multivariable analysis because the risk factors of interest were identified a priori.

To address the exploratory objective, a Spearman’s correlation test was conducted to test the relationships between the socio-economic variables. Mediation analyses were performed to examine the mediating effects of socio-environmental factors on neurocognitive and behavioral outcomes. To ensure that the model was meaningful and to reduce redundancy, only variables that were significantly associated with outcomes in the multivariable models and those deemed to be conceptually relevant based on consensus from the investigators were included in the mediation analyses. Mediation pathways were tested using the PROCESS algorithm for SPSS (55). Separate mediation models were run for each outcome measure. Survivors’ current age, sex, age at diagnosis and CRT status were included as covariates within mediation models. Unstandardized point estimates and bootstrapped 95% CIs (BCCI) for the total indirect effect and specific indirect pathways were estimated. Missing data were handled using listwise deletion. All analyses were conducted using SPSS version 26 (Chicago, SPSS Inc.). A *P* value <.05 was considered statistically significant, and all statistical tests were two-sided.

## Results

### Comparison Between CRT Group Versus Non-CRT Group

A minority of survivors (n = 32, 21.1%) received CRT; the others were treated with chemotherapy-only protocols (**Supplement 3**). There were more male survivors in the CRT group than in the non-CRT group (78.1% vs 45.0%, *P* <.001). On average, survivors in the CRT group were older (30.8 [6.8] vs 21.5 [6.0] years, *P* <.0001) and had survived for a longer time since diagnosis (24.0 [7.9] vs 15.4 [6.4] years, *P* <.0001) than those in the non-CRT group. The CRT group also had significantly higher proportions of survivors who were diagnosed with an endocrine (12.5% vs 2.5%, *P* = 0.016), metabolic (12.5% vs 2.5%, *P* = 0.016) or neurological (18.8% vs 3.3%, *P* = 0.002) condition, compared with the non-CRT group.

### Socio-Environmental Factors

The socio-environmental characteristics of the study cohort are presented in **Supplement 4**. The mean CUHK-PARCY score for physical activity was 6.0 (SD = 1.6, range 1 = 10), indicating that survivors generally participated in moderate physical activities for durations > 20 minutes once or twice per week. The survivors’ self-reported fatigue score was 68.3 (SD = 14.8, range = 34.7 – 100). The mean family functioning score was 68.1 (SD = 21.4, range = 33 – 172). A slight majority (n = 90, 59.2%) of the survivors resided in less than 600 square feet of living space.

### Neurocognitive and Behavioral Outcomes

**Figure 1:**
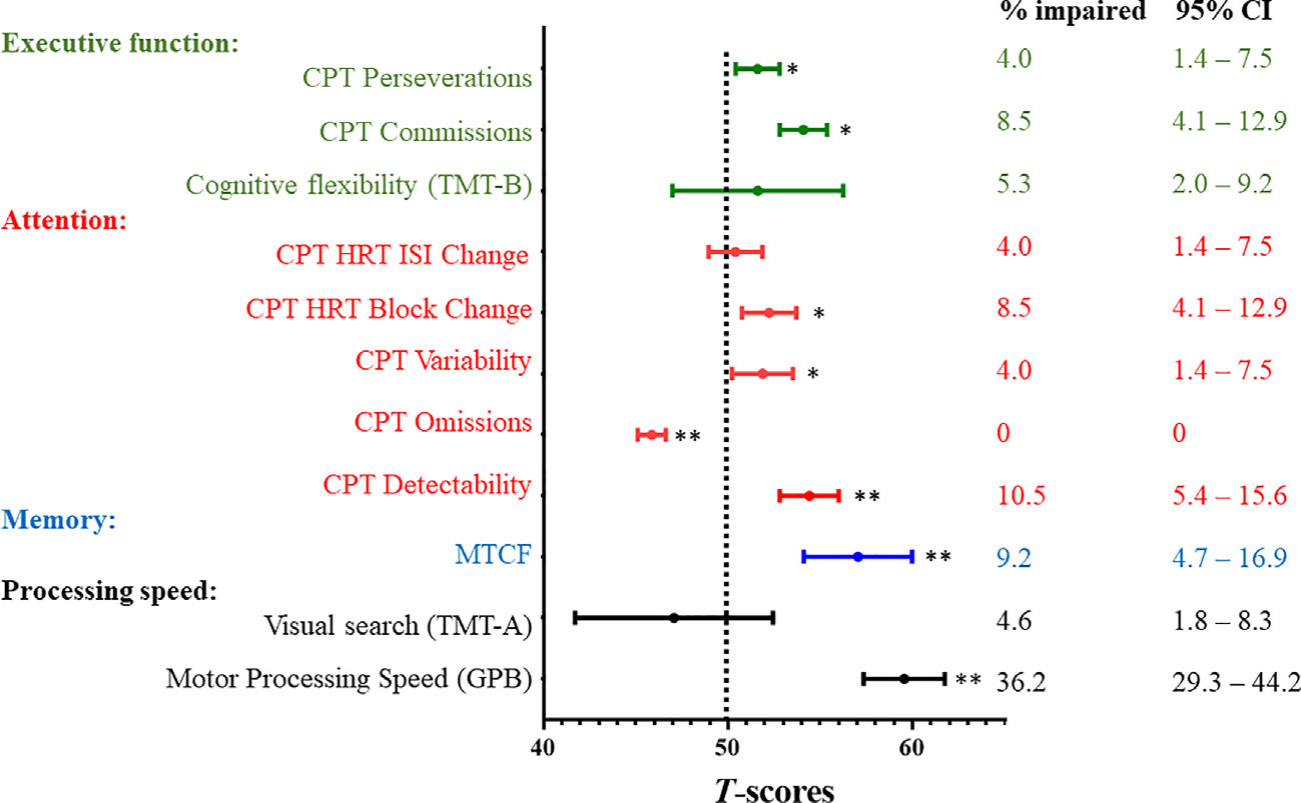
Neurocognitive Outcomes and Prevalence Rates of Impairment. CI, confidence interval; CPT, Conners Continuous Performance Test-III; GPB, Grooved Pegboard; HRT, hit reaction time; ISI, inter-stimulus Intervals; MTCF, Modified Taylor Complex Figure; SD, standard deviation; TMT, Trail Making Test. All neurocognitive measures were transformed into age-adjusted *T*-scores (mean = 50; standard deviation [SD] = 10) using references provided by the test manuals or the published literature (**Supplement 1**). All *T*-scores were scaled such that a higher score was indicative of worse functioning. A one-sample *t* test was used to compare the survivors’ performance with population norms (Dotted line; *T*-score = 50). * indicates statistical significance at *P*≦0.05 after correcting for false discovery rate ** indicates statistical significance at *P*≦0.01 after correcting for false discovery rate To estimate the prevalence of impairments within the study sample, impairment was defined as a score below the 1.5 standard deviation poorer than the age-adjusted *T*-scores of reference norms. Prevalence estimates are expressed as proportion (%) and 95% confidence intervals.

Survivors performed more poorly than the reference norms on measures of motor processing speed, memory, executive function, and attention after correcting for false discovery rate (all *P* values <.05; **Figure 1**). The mean scores of all cognitive measures are presented in **Supplement 5**. A minority of survivors demonstrated impairments in memory (9.2%), motor processing speed (36.2%) and executive function on CPT Commissions (8.5%) (**Figure 1**). The rates of impairment on attention measures ranged from 4.0% to 10.5% (**Figure 1**).

**Figure 2:**
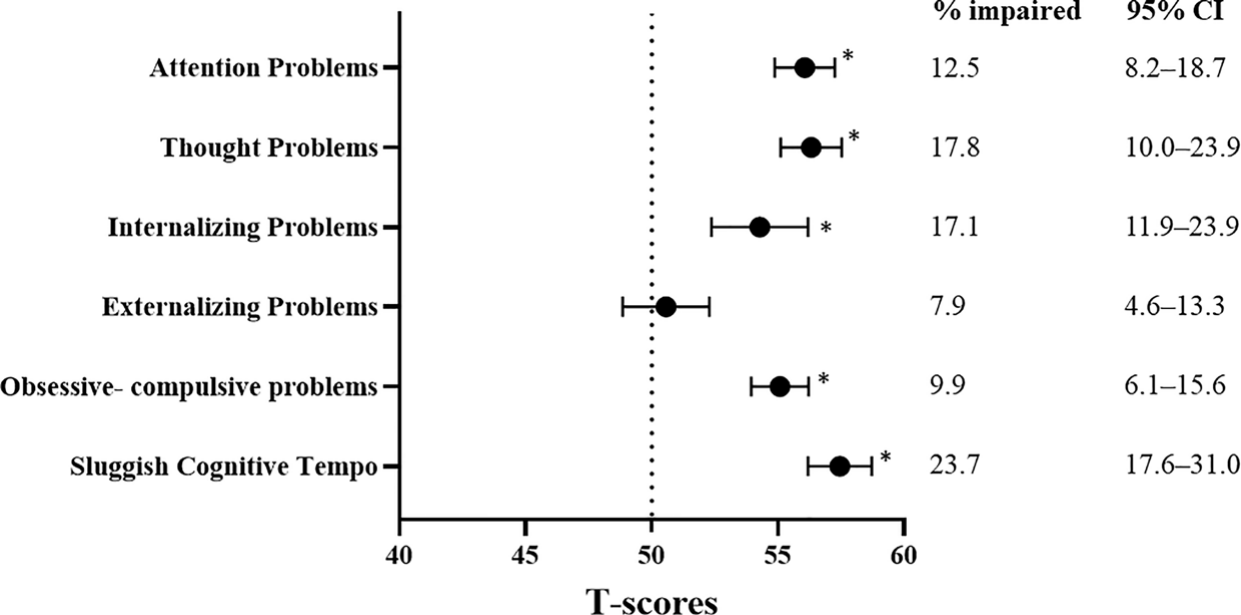
Behavioral Outcomes and Prevalence Rates of Impairment. CI, confidence interval; SD, standard deviation. All behavioral measures were transformed into age-adjusted *T*-scores (mean = 50; standard deviation [SD] = 10) using references provided by the test manuals or the published literature (**Supplement 1**). All *T*-scores were scaled such that a higher score was indicative of more problems. A one-sample *t* test was used to compare the survivors’ performance with population norms (Dotted line: *T*-score = 50). * indicates statistical significance at *P*≦0.01 after correcting for false discovery rate. To estimate the prevalence of impairments within the study sample, impairment was defined as a score below the 1.5 standard deviation poorer than the age-adjusted *T*-scores of reference norms. Prevalence estimates are expressed as proportion (%) and 95% confidence intervals.

Compared with the age- and sex-matched norms, the survivors reported significantly more issues with all measures of behavioral functioning (all *P* values <.001; **Figure 2**). The mean scores of all behavioral measures are presented in **Supplement 5**. On the syndrome scales, the proportions of survivors who reported symptoms of inattention and internalizing and externalizing problems ranged from 7.9% to 17.1%. Approximately a fifth of survivors reported thought problems (17.8%), and nearly a quarter reported a sluggish cognitive tempo (23.7%).

Survivors in the CRT group demonstrated worse cognitive flexibility on TMT-B than those in the non-CRT group (mean [SD] 60.7 [28.2] vs 48.9 [20.9], *P* = .050) (**Supplement 6**). The CRT group reported more obsessive-compulsive problems (*P* = 0.048) and anxiety problems (*P* = 0.015) than the non-CRT group (**Supplement 6**). There were no between-group differences in the other neurocognitive and behavioral domain scores. Descriptively, the impairment rates for internalizing problems (31.3% vs 13.3%), externalizing problems (12.5% vs 6.7%) and obsessive-compulsive problems (18.8% vs 7.5%) were almost twice as high in the CRT group as in the non-CRT group.

### Factors Associated With Neurocognitive Outcomes

After adjusting for sex, age at diagnosis, age at evaluation and CRT status, multivariable analysis showed that survivors who developed chronic health conditions had more executive dysfunction than those who did not have any chronic conditions (CPT commission: B = 5.09, SE = 2.05, *P* = .014) (**Table 2**). Interestingly, higher number of intrathecal chemotherapy injections was associated with a better performance on executive function (CPT commission: B = -0.024, SE = 0.09, *P* = .049, **Table 2**).

**Table 2:** Factors Associated with Neurocognitive Outcomes.

Risk factors*	Motor processing speed (Grooved Pegboard)^			Memory (MTCF)^			Inattentiveness (CPT Detectability)^			Inattentiveness (CPT Commissions)^			Sustained attention (CPT HRT block change)^		
	B	SE	*P*	B	SE	*P*	B	SE	*P*	B	SE	*P*	B	SE	*P*
**Treatment factors**															
**Risk group**															
**Intermediate/high risk**	0.96	2.4	0.68	1.19	3.2	0.71	2.39	1.77	0.18	0.62	1.59	0.70	0.97	1.76	0.58
**Standard risk (referent)**	–	–	–	–	–	–	–	–	–	–	–	–	–	–	–
**IV HDMTX ^Ɨ^**															
**20 g/m^2^**	2.14	3.37	0.53	6.42	4.59	0.17	0.39	2.31	0.86	1.98	2.54	0.44	2.83	2.22	0.21
**8 g/m^2^ (referent)**	–	–	–	–	–	–	–	–	–	–	–	–	–	–	–
**IT chemotherapy (no. of injections)**	0.005	0.34	0.98	0.14	0.47	0.75	0.20	0.25	0.43	-0.024	0.09	**0.049**	-0.039	0.02	0.052
**Chronic health conditions**															
**Any**	4.94	2.75	0.074	7.87	3.98	0.051	2.94	2.01	0.14	5.09	2.05	**0.014**	-0.49	1.83	0.78
**No (referent)**	–	–	–	–	–	–	–	–	–	–	–	–	–	–	–
**Socio-environmental factors**															
**Living space^※^**															
**≤ 600 square feet**	4.75	2.21	**0.034**	6.50	2.88	**0.027**	1.96	1.68	0.24	1.15	1.75	0.51	1.41	1.53	0.36
**>600 square feet (referent)**	–	–	–	–	–	–	–	–	–	–	–	–	–	–	–
**Family functioning^**	0.06	0.05	0.23	-0.09	0.06	0.16	0.04	0.04	0.34	0.07	0.04	**0.049**	-0.02	0.03	0.53
**Physical activity#**	0.49	0.75	0.52	0.83	0.95	0.38	-0.69	0.53	0.19	-1.05	0.55	0.059	0.08	0.48	0.85
**Fatigue#**	-0.05	0.07	0.55	-0.14	0.09	0.14	-0.09	0.05	0.13	-0.11	0.05	0.070	0.06	0.05	0.20
**Academic stress^^,§^**	0.10	0.16	0.53	0.09	0.21	0.65	-0.07	0.09	0.48	-0.04	0.11	0.72	0.15	0.11	0.17

B, unstandardized coefficient; CPT, Conners Continuous Performance Test; HDMTX, high-dose methotrexate; HRT, hit reaction time; IT, intrathecal; IV, intravenous; MTCF, Modified Taylor Complex Figure; SE, standard error.

*All statistical models were adjusted for sex, age at diagnosis, age at evaluation and cranial irradiation. Only measures on which the survivors differed from the normative samples after applying false discovery rate, and had an impairment rate of more than 5% (**Figure 2**) were examined for associations with risk factors. Boldface indicates statistical significance at P≦0.05.

^^^A higher value was indicative of worse functioning.

^#^A higher value was indicative of better functioning.

^Ɨ^Total HDMTX categorized as 8 g/m^2^ (4 cycles of 2 g/m^2^) of methotrexate versus 20 g/m^2^ (4 cycles of 5 g/m^2^).

^§^Academic stress was evaluated in survivors who were still schooling.

Survivors who resided in < 600 square feet of housing space had worse performances in the domains of memory (B = 6.50, SE = 2.88; *P* = .027) and motor processing speed (B = 4.75, SE = 2.21; *P* = .034) than survivors who resided in larger housing spaces. Worse family functioning was associated with more executive dysfunction (CPT commission: B = 0.07, SE = 0.04, *P* = .049). No associations of neurocognitive outcomes with other socio-environmental variables were observed.

### Factors Associated With Behavioral Outcomes

Compared with survivors without chronic health conditions, those who developed chronic health conditions reported more symptoms of inattention (B = 5.75, SE = 1.43, *P* <.0001) and internalizing (B = 5.99, SE = 2.40, *P* = .014), externalizing problems (B = 4.62, SE = 2.16, *P* = .034), and sluggish cognitive tempo (B = 5.34, SE = 1.56, *P* = .001; **Table 3**). Survivors who were treated with a cumulative dose of 20 g/m^2^ of HDMTX had more externalizing problems than survivors treated with 8 g/m^2^ of HDMTX (B = 5.31, SE = 2.36, *P* = .027).

**Table 3:** Factors Associated with Behavioral Outcomes.

Risk factors*	**Attention problems^**			**Thought problems^**			**Internalizing problems^**			**Externalizing problems^**			**Obsessive- compulsive problems^**			**Sluggish cognitive tempo^**		
	B	SE	*P*	B	SE	*P*	B	SE	*P*	B	SE	*P*	B	SE	*P*	B	SE	*P*
**Treatment factors**																		
**Risk group**																		
**Intermediate/high risk**	0.32	1.25	0.79	0.19	1.24	0.87	0.18	2.09	0.92	0.43	1.87	0.81	1.57	1.19	0.19	0.18	1.40	0.89
**Standard risk (referent)**	–	–	–	–	–	–	–	–	–	–	–	–	–	–	–	–	–	–
**IV HDMTX ^Ɨ^**																		
**20 g/m^2^**	0.78	1.53	0.61	0.65	1.71	0.70	2.96	2.72	0.27	5.31	2.36	0.027	0.99	1.65	0.55	1.05	1.83	0.56
**8 g/m^2^ (referent)**	–	–	–	–	–	–	–	–	–	–	–	–	–	–	–	–	–	–
**IT chemotherapy (no. of injections)**	0.002	0.01	0.89	0.005	0.01	0.73	-0.02	0.02	0.37	-0.04	0.01	**0.042**	-0.01	0.01	0.27	-0.015	0.01	0.35
**Chronic health conditions**																		
**Any**	5.73	1.43	**<0.0001**	3.05	1.53	**0.048**	5.99	2.40	**0.014**	4.62	2.16	**0.034**	1.12	1.43	0.43	5.34	1.56	**0.001**
**No (referent)**	–	–	–	–	–	–	–	–	–	–	–	–	–	–	–	–	–	–
**Socio-environmental factors**																		
**Living space^※^**																		
**≤ 600 square feet**	1.91	1.23	0.12	0.79	1.27	0.53	4.06	1.99	**0.044**	0.29	1.81	0.87	0.46	1.18	0.69	2.89	1.31	**0.030**
**>600 square feet** **(referent)**	–	–	–	–	–	–	–	–	–	–	–	–	–	–	–	–	–	–
**Family functioning^**	0.14	0.03	**<0.0001**	0.15	0.03	**<0.0001**	0.29	0.04	**<0.0001**	0.23	0.04	**<0.0001**	0.13	0.03	**<0.0001**	0.15	0.03	**<0.0001**
**Physical activity#**	-1.12	0.38	**0.004**	-0.87	0.39	**0.030**	-1.04	0.63	0.10	-0.71	0.57	0.21	-0.60	0.37	0.10	-1.22	0.41	**0.003**
**Fatigue#**	-0.30	0.03	**<0.0001**	-0.28	0.04	**<0.0001**	-0.53	0.05	**<0.0001**	-0.38	0.05	**<0.0001**	-0.24	0.03	**<0.0001**	-0.35	0.03	**<0.0001**
**Academic stress^^,§^**	0.21	0.08	**0.017**	0.16	0.09	0.08	0.49	0.13	**<0.0001**	0.38	0.11	**0.002**	0.19	0.08	**0.028**	0.19	0.09	**0.044**

B, unstandardized coefficient; HDMTX, high-dose methotrexate; IT, intrathecal; IV, intravenous; SE, standard error.

*All statistical models were adjusted for sex, age at diagnosis, age at evaluation and cranial radiation. Boldface indicates statistical significance at P≦0.05.

^^^A higher value was indicative of worse functioning. Refer to **Supplement 2** on detailed explanation of specific behavioral domains.

^#^A higher value was indicative of better functioning.

^Ɨ^Total HDMTX categorized as 8 g/m^2^ (4 cycles of 2 g/m^2^) of methotrexate versus 20 g/m^2^ (4 cycles of 5 g/m^2^).

^§^Academic stress was evaluated in survivors who were still schooling.

Regarding the living environment, poorer family functioning was strongly associated with all measures on the syndrome scales (**Table 3**). Survivors who resided in < 600 square feet of housing space were more likely to report internalizing problems (B = 4.06, SE = 1.99, *P* = .044) and a sluggish cognitive tempo (B = 2.89, SE = 1.31, *P* = .030) than survivors with larger housing spaces.

A higher level of physical inactivity was correlated with more self-reported symptoms of inattention (B = -1.12, SE = 0.38, *P* = .004) and a sluggish cognitive tempo (B = -1.22, SE = 0.41, *P* = .003; **Table 3**). Survivors who were more fatigued reported significantly worse outcomes on all measures of the syndrome scale (all *P* values <.0001). Among the subset of survivors who were current students, academic stress was associated with multiple domains of behavioral functioning (all *P* values <.05).

### Exploratory Analysis

A positive correlation was also observed between physical inactivity and fatigue (r = 0.34, *P* <.0001), and strong inter-correlations were identified between fatigue, family functioning and academic stress (**Supplement 8**).

**Figure 3:**
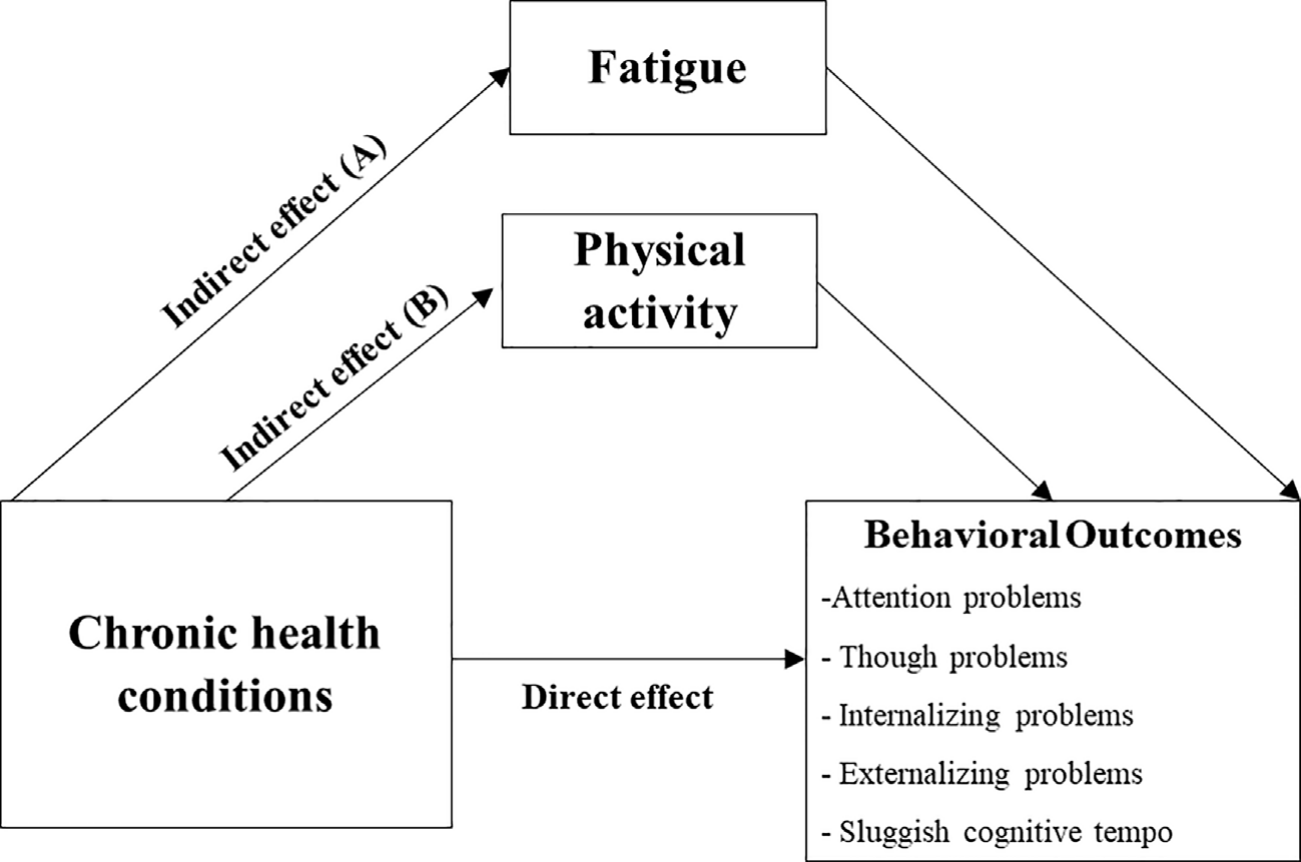
Predicted Mediation Pathway (Exploratory Analysis). Predicted mediation pathway for association between chronic health conditions and behavioral outcomes, *via* fatigue **(A)** and physical activity **(B)**. Mediation analysis was run separately for each behavioral measure, controlling for sex, age at evaluation, age at diagnosis and cranial radiation.

Based on findings from the primary analyses and consensus from the investigators, we explored the mediating effect of fatigue on the relationship between chronic health conditions and self-reported behavioral outcomes, adjusting for current age, age at diagnosis and CRT status (**Figure 3A**). The analysis was then repeated, replacing fatigue with physical activity as the mediator (**Figure 3B**).

Chronic health conditions were significantly associated with self-reported attention problems indirectly through fatigue (ß = 3.06, *P* = 0.007; total indirect effect ß = 3.12, 95% BCCI 1.27 – 5.81). Similar results were obtained for other behavioral measures on the syndrome scales, except for thought problems (**Supplement 9**). Physical activity was not identified as a mediator between chronic health conditions and behavioral outcomes. Unstandardized point estimates and 95% BCCIs for the total indirect effect and specific indirect pathways are summarized in **Supplement 9**.

## Discussion

This is one of the first studies to evaluate the neurocognitive and behavioral outcomes of Chinese survivors of childhood ALL. A minority of survivors (4.0% to 36.2%) demonstrated moderate impairment on performance-based cognitive assessments across the domains of attention, executive function, motor processing speed and memory. Our results are consistent with those of studies in which ALL survivors were shown to exhibit deficits in these domains (4, 7, 26). Survivors reported more behavioral problems relative to the population norms, particularly in the domains of attention, a sluggish cognitive tempo and depressive symptoms.

Consistent with the literature (7, 15, 22), we found that the CRT group displayed significantly worse memory performances than the non-CRT group. The lack of differences in other neurocognitive and behavioral scores between the CRT and non-CRT survivors is probably due to the limited sample size in the former group. However, the rates of deficits in internalizing and emotional problems were descriptively higher in the CRT group. Specifically, a third of the survivors in the CRT group reported internalizing problems, such as depression, anxiety and withdrawal behaviors. Brain injuries induced by the cancer itself or by neurotoxic treatments, such as CRT, at an early stage in life can severely affect critical periods of brain development (7). Beyond the direct neurotoxic effect of CRT, the effects of aging and CRT-associated chronic health conditions may have contributed to the higher rates of functional impairment in the CRT group (15, 22). Hence, our results are consistent with well-established evidence indicating that survivors exposed to CRT face the highest burden of morbidity and require intensive cognitive and behavioral rehabilitation.

Fortunately, most contemporary international treatment protocols for ALL have eliminated CRT. Collective evidence from the literature on long-term survivors demonstrates the probable cognitive-sparing effect of chemotherapy-based protocols over CRT (4, 7, 8, 31, 56). However, survivors still suffer from apparent cognitive impairment due to the administration of intensive chemotherapy.

Although methotrexate is an integral component of modern ALL treatment regimen with known neurotoxic effects, we did not identify a significant and consistent dose-dependent relationship between HDMTX and long-term outcomes. Possibly, the cumulative dose is a less sensitive surrogate for drug exposure, as it may introduce errors of measurement and compromise the accuracy of the results (6). We also did not manage to account for the psychological effect of leucovorin rescue in this retrospective study. Interestingly, the use of IT chemotherapy was associated with better outcomes, probably because IT chemotherapy is a common component of contemporary treatment regimens and is typically associated with better functional outcomes than traditional CRT-based therapies in earlier eras. This probably explained why further post-hoc analysis showed that the association between IT chemotherapy and neurocognitive outcomes were no longer significant when the analysis was conducted only within the non-CRT group. Our future work will include the prospective collection of plasma HDMTX exposure and leucovorin rescue data as a more precise approach to identify the sources of long-term neurocognitive outcomes.

The association of specific treatment exposures at an early stage of life with health morbidities over time has been well established in survivors of childhood cancer, particularly in CRT survivors (20, 21). Our cohort of Chinese survivors was still relatively young. Still, nearly a fifth of the non-CRT survivors had developed clinical cardiopulmonary complications, psychiatric disorders and other health conditions. Notably, these chronic health conditions were associated with worse outcomes on multiple self-reported behavioral measures, even after accounting for CRT status. This finding is similar to those of emerging studies that identified the contributions of chronic health conditions resulting from childhood cancer therapies to emotional distress and neurocognitive impairment (22, 23, 25). These observations have important clinical implications. First, although the chronic health conditions in these survivors may be irreversible, the timely treatment of late effects may help to alleviate the psychological and behavioral symptoms. Hence, our findings emphasize the importance of systematic screening for chronic health conditions in survivors; to the best of our knowledge, this is not commonly practiced at most institutions in China (32). Second, the routine monitoring of psychological symptoms in young survivors who have developed chronic health conditions may facilitate the early identification of behavioral problems before they develop into clinical developmental problems and affect functional outcomes.

Our results offer valuable new insights regarding culturally relevant socio-environmental factors that influence behavioral functioning in Chinese survivors of ALL. We found that a smaller living space was associated with worse memory and motor processing speed performances on performance-based measures. Poorer family functioning correlated with more self-reported behavioral problems. General population studies have shown that environmental characteristics, such as noise pollution, overcrowding and housing problems, can affect neurodevelopment in children (57–59). Although we did not have data on the household size and living space per person, this finding is still especially relevant to the context of Hong Kong, where the type, area and size of housing are surrogate markers of socio-economic status and access to quality healthcare (60, 61). Also notable is that the assessments of survivors in this study were conducted in 2019, during which Hong Kong was affected by citywide social unrest. Increases in family conflicts and emotional distress were reported during that period (62). Similarly, academic stress is widespread in most Asian societies, particularly in Chinese cultures (63, 64). From a service perspective, our findings highlight the importance of identifying these potentially modifiable risk factors and developing culturally relevant preventive interventions (e.g., providing social support to improve the family environment, mindfulness interventions to relieve academic stress) to improve the clinical and psychosocial outcomes of survivors.

Consistent with the literature (54, 65), in our study, fatigue was consistently associated with multiple measures of behavioral functioning. The survivors’ self-reported fatigue score (mean = 68.3 points) corresponded to a mild-to-moderate level of fatigue, based on the published thresholds in individuals with major conditions (66, 67). Particularly, the results of our exploratory pathway analysis support the mediating effect of fatigue on the relationship between chronic health conditions and behavioral outcomes. Although physical activity was not a significant mediator of this relationship, it is strongly associated with both fatigue and measures of behavior functioning. Taken together, these preliminary findings complement the growing evidence in the literature that supports the beneficial effects of physical activity on mental health in cancer survivors (65, 68–70). From a physiological perspective, neurobehavioral changes are closely related to aging in the general population (71). Exercise may potentially delay the onset of premature frailty and chronic disease burdens and could lead to better psychosocial outcomes in survivors (72, 73). From a psychological perspective, certain types of sports promote teamwork and self-efficacy. The emotional and educational influences of physical activities may address specific externalizing problems in adolescents, such as rule-breaking behaviors, aggression and antisocial personality traits (74). For example, adventure-based and family-centered physical activity programs have been shown to reduce fatigue, depression and anxiety in survivors in both domestic and international studies (68, 75, 76).

Our study has a few limitations. This single-center study recruited survivors through non-probability sampling, which may have been subject to sampling bias. However, a post-hoc analysis did not identify major differences between participants and non-participants, except for a marginal older age at follow-up for the non-participant group (**Supplement 10**). The population of childhood ALL survivors in Hong Kong is small and we attempted to recruit every eligible survivor from consecutive sampling at the LTFU clinic; this approach would have established a reasonable sampling frame. While we acknowledge that the treatment protocols represented in our study cohort are heterogeneous and span across 1990s to 2000s, the treatment agents (except CRT) remain the backbone of modern therapies for childhood ALL. The treatment strategies across the eras are no different from protocols captured in epidemiological studies of other countries (22, 77–81). By including survivors who were treated in the earlier eras, our cohort is representative of the growing population of aging cancer survivors in the current health care system. We did not include a control group of subjects without cancer for comparison. However, the rates of impairment presented in this study were based on reference norms (Chinese norms for TMT-A, TMT-B and behavioral assessments), and our data do suggest that the survivors demonstrated worse behavioral outcomes relative to the general population. Although our sample size was calculated *a priori* to detect differences in cognitive outcomes between survivors and norms, it may not be sufficiently powered to detect associations with multiple risk factors. However, our findings are consistent with the robust literature that identifies CRT, fatigue and chronic health conditions as poor predictors of functioning. A minority of survivors in our sample were diagnosed before 2000, or prior to the implementation of computerized medical records, and therefore had incomplete medical and treatment records. For example, the total CRT doses and cumulative doses of most chemotherapy drugs could not be calculated because of incomplete documentation. Furthermore, a common source bias might explain the strong associations between self-reported behavioral outcomes and socio-environmental factors.

Future studies should include a more objective evaluation of variables, such as actigraphy studies physical activity and sleep, as well as culturally relevant measures of social attainment outcomes (e.g. housing tenure, employment history, personal income level etc.). Despite these limitations, this work serves as both a feasibility study and a model with which to facilitate larger-scale research that will validate our preliminary findings. To the best of our knowledge, this is one of the largest studies to examine the long-term neurocognitive and psychosocial outcomes of Chinese survivors of childhood ALL. A multicenter study that involves the prospective collection of outcome data and comparison with an age- and sex- matched control group may also better reflect the trajectories of functional outcomes in these Chinese survivors as they advance from early to long-term survivorship.

## Conclusion

Our findings suggest that the majority of young survivors of ALL exhibit normal cognitive and behavioral function during the early phase of survivorship. However, subgroups of survivors who developed chronic health conditions or were exposed to adverse socio-environmental conditions were found to be at risk of developing poor functional outcomes. These individuals require closer pre-emptive screening to identify behavioral and cognitive problems before they develop into clinical developmental conditions that could impair the survivors’ educational and occupational outcomes. We acknowledge that it may not be possible to fully eliminate the neurotoxic effects of chemotherapy. Nevertheless, survivors may benefit from appropriate interventions to address modifiable risk factors, such as the provision of social support to families and interventions to encourage survivors to adopt a physically active lifestyle. Our findings should be validated in a larger-scale study that involves the prospective collection of outcome data, as this would better reflect the trajectories of neurocognitive and behavioral changes in survivors of childhood ALL in Hong Kong. We also expect that multinational and collaborative trials might facilitate comprehensive investigations of racial/ethnic-specific outcomes and the associated risk factors.

## Data Availability Statement

The raw data supporting the conclusions of this article will be made available by the authors, upon reasonable request.

## Ethics Statement

The studies involving human participants were reviewed and approved by Joint Chinese University of Hong Kong – New Territories East Cluster Clinical Research Ethics Committee. Written informed consent to participate in this study was provided by the participants’ legal guardian/next of kin.

## Author Contributions

Conception or design of the work: All. Data acquisition: LP, PY, LY, YTC. Data analysis: LP, PY, YTC. Data interpretation: All. Drafting of report: LP, PY, YTC. Revising it critically for important intellectual content: All. All authors contributed to the article and approved the submitted version. Agreement to be accountable for all aspects of the work: All.

## Funding

This study is funded by the Hong Kong Research Grant Council (ref no: 24614818), awarded to YTC.

## Conflict of Interest

The authors declare that the research was conducted in the absence of any commercial or financial relationships that could be construed as a potential conflict of interest.

## References

[1] Pui CH. Evans WE. A 50-year journey to cure childhood acute lymphoblastic leukemia. Semin Hematol (2013) 50(3):185–96. 10.1053/j.seminhematol.2013.06.007 PMC3771494 23953334

[2] Bhakta N Liu Q Ness KK Baassiri M Eissa H Yeo F. The cumulative burden of surviving childhood cancer: An initial report from the St Jude Lifetime Cohort Study (SJLIFE). Lancet (2017) 390(10112):2569–82. 10.1016/S0140-6736(17)31610-0 PMC5798235 28890157

[3] Landier W Skinner R Wallace WH Hjorth L Mulder RL Wong FL. Surveillance for late effects in childhood cancer survivors. J Clin Oncol (2018) 36(21):2216–22. 10.1200/JCO.2017.77.0180 PMC6804892 29874139

[4] Cheung YT Krull KR. Neurocognitive outcomes in long-term survivors of childhood acute lymphoblastic leukemia treated on contemporary treatment protocols: A systematic review. Neurosci Biobehav Rev (2015) 53:108–20. 10.1016/j.neubiorev.2015.03.016 PMC4425605 25857254

[5] Iyer NS Balsamo LM Bracken MB Kadan-Lottick NS. Chemotherapy-only treatment effects on long-term neurocognitive functioning in childhood ALL survivors: A review and meta-analysis. Blood (2015) 126(3):346–53. 10.1182/blood-2015-02-627414 26048910

[6] Krull KR Cheung YT Liu W Fellah S Reddick WE Brinkman TM. Chemotherapy pharmacodynamics and neuroimaging and neurocognitive outcomes in long-term survivors of childhood acute lymphoblastic leukemia. J Clin Oncol (2016) 34(22):2644–53. 10.1200/JCO.2015.65.4574 PMC5321052 27269941

[7] Krull KR Brinkman TM Li C Armstrong GT Ness KK Srivastava DK. Neurocognitive outcomes decades after treatment for childhood acute lymphoblastic leukemia: A report from the St Jude Lifetime Cohort Study. J Clin Oncol (2013) 31(35):4407–15. 10.1200/JCO.2012.48.2315 PMC3842908 24190124

[8] Godoy PBG Simionato NM de Mello CB Suchecki D. Assessment of executive functions after treatment of childhood acute lymphoid leukemia: A systematic review. Neuropsychol Rev (2020) 30(3):386–406. 10.1007/s11065-020-09446-4 32720195

[9] Cheung YT Sabin ND Reddick WE Bhojwani D Liu W Brinkman TM. Leukoencephalopathy and long-term neurobehavioural, neurocognitive, and brain imaging outcomes in survivors of childhood acute lymphoblastic leukaemia treated with chemotherapy: A longitudinal analysis. Lancet Haematol (2016) 3(10):e456–66. 10.1016/S2352-3026(16)30110-7 PMC5417340 27658980

[10] Liu W Cheung YT Brinkman TM Banerjee P Srivastava D Nolan VG. Behavioral symptoms and psychiatric disorders in child and adolescent long-term survivors of childhood acute lymphoblastic leukemia treated with chemotherapy only. Psycho-Oncology (2018) 27(6):1597–607. 10.1002/pon.4699 PMC5986588 29521470

[11] Anestin AS Lippé S Robaey P Bertout L Drouin S Krajinovic M. Psychological risk in long-term survivors of childhood acute lymphoblastic leukemia and its association with functional health status: A PETALE cohort study. Pediatr Blood Cancer (2018) 65(11):e27356. 10.1002/pbc.27356 30084222

[12] Ferguson WS. School performance in childhood leukemia survivors. J Pediatr (2019) 205:2–3. 10.1016/j.jpeds.2018.12.018 30684977

[13] Huang I Brinkman TM Cheung YT Pui C Hudson MM Krull KR. Functional consequence of cognitive impairment in survivors of childhood acute lymphoblastic leukemia (ALL): The role of cancer symptoms as mediators. J Clin Oncol (2016) 34(3):235. 10.1200/jco.2016.34.3_suppl.235 26573075

[14] Phillips NS Cheung YT Glass JO Scoggins MA Liu W Ogg RJ. Neuroanatomical abnormalities related to dexamethasone exposure in survivors of childhood acute lymphoblastic leukemia. Pediatr Blood Cancer (2020) 67(3):e27968. 10.1002/pbc.27968 31407461 PMC6980878

[15] Williams AM Cheung YT Hyun G Liu W Ness KK Ehrhardt MJ. Childhood neurotoxicity and brain resilience to adverse events during adulthood. Ann Neurol (2021) 89(3):534–45. 10.1002/ana.25981 PMC7897299 33274777

[16] van der Plas E Nieman BJ Butcher DT Hitzler JK Weksberg R Ito S. Neurocognitive late effects of chemotherapy in survivors of acute lymphoblastic leukemia: Focus on methotrexate. J Can Acad Child Adolesc Psychiatry (2015) 24(1):25–32. 26336377 PMC4357331

[17] Cheung YT Eskind A Inaba H Hudson MM Pui C Krull KR. Association of bacteremic sepsis with long-term neurocognitive dysfunction in pediatric patients with acute lymphoblastic leukemia. JAMA Pediatr (2018) 172(11):1092–5. 10.1001/jamapediatrics.2018.2500 PMC6248158 30264151

[18] Cheung YT Khan RB Liu W Brinkman TM Edelmann MN Reddick WE. Association of cerebrospinal fluid biomarkers of central nervous system injury with neurocognitive and brain imaging outcomes in children receiving chemotherapy for acute lymphoblastic leukemia. JAMA Oncol (2018) 4(7):e180089. 10.1001/jamaoncol.2018.0089 29596541 PMC5885182

[19] Sabin ND Cheung YT Reddick WE Bhojwani D Liu W Glass JO. The impact of persistent leukoencephalopathy on brain white matter microstructure in long-term survivors of acute lymphoblastic leukemia treated with chemotherapy only. AJNR Am J Neuroradiol (2018) 39(10):1919–25. 10.3174/ajnr.A5791 PMC6432797 30213807

[20] Oeffinger KC Mertens AC Sklar CA Kawashima T Hudson MM Meadows AT. Chronic health conditions in adult survivors of childhood cancer. N Engl J Med (2006) 355(15):1572–82. 10.1056/NEJMsa060185 17035650

[21] Hudson MM Ness KK Gurney JG Mulrooney DA Chemaitilly W Krull KR. Clinical ascertainment of health outcomes among adults treated for childhood cancer. JAMA (2013) 309(22):2371–81. 10.1001/jama.2013.6296 PMC3771083 23757085

[22] Cheung YT Brinkman TM Li C Mzayek Y Srivastava D Ness KK. Chronic health conditions and neurocognitive function in aging survivors of childhood cancer: A report from the Childhood Cancer Survivor Study. J Natl Cancer Inst (2018) 110(4):411–41. 10.1093/jnci/djx224 PMC6059140 29088360

[23] Vuotto SC Krull KR Li C Oeffinger KC Green DM Patel SK. Impact of chronic disease on emotional distress in adult survivors of childhood cancer: A report from the childhood cancer survivor study. Cancer (2017) 123(3):521–8. 10.1002/cncr.30348 PMC5258824 27764524

[24] van der Plas E Qiu W Nieman BJ Yasui Y Liu Q Dixon SB. Sex-specific associations between chemotherapy, chronic conditions and neurocognitive impairment in ALL survivors: A report from the Childhood Cancer Survivor Study. J Natl Cancer Inst (2020)3:djaa136. 10.1093/jnci/djaa136 PMC8096369 32882041

[25] Bass JK Liu W Banerjee P Brinkman TM Mulrooney DA Gajjar A. Association of hearing impairment with neurocognition in survivors of childhood cancer. JAMA Oncol (2020) 6(9):1363–71. 10.1001/jamaoncol.2020.2822 PMC7393588 32729886

[26] Peng L Yam PP Yang LS Sato S Li CK Cheung YT. Neurocognitive impairment in Asian childhood cancer survivors: A systematic review. Cancer Metastasis Rev (2020) 39(1):27–41. 10.1007/s10555-020-09857-y 31965433

[27] Syn NL Yong WP Lee SC Goh BC. Genetic factors affecting drug disposition in asian cancer patients. Expert Opin Drug Metab Toxicol (2015) 11(12):1879–92. 10.1517/17425255.2015.1108964 26548636

[28] Park DC Huang C. Culture wires the brain: A cognitive neuroscience perspective. Perspect Psychol Sci (2010) 5(4):391–400. 10.1177/1745691610374591 22866061 PMC3409833

[29] Choudhury S. Culturing the adolescent brain: What can neuroscience learn from anthropology? Soc Cognit Affect Neurosci (2010) 5(2-3):159–67. 10.1093/scan/nsp030 PMC2894667 19959484

[30] Ho C Bluestein DN Jenkins JM. Cultural differences in the relationship between parenting and children’s behavior. Dev Psychol (2008) 44(2):507–22. 10.1037/0012-1649.44.2.507 18331140

[31] Krull KR Hardy KK Kahalley LS Schuitema I Kesler SR. Neurocognitive outcomes and interventions in long-term survivors of childhood cancer. J Clin Oncol (2018) 36(21):2181–9. 10.1200/JCO.2017.76.4696 PMC6553837 29874137

[32] Poon L Yu C Peng L Ewig C Zhang H Li C. Clinical ascertainment of health outcomes in Asian survivors of childhood cancer: A systematic review. J Cancer Surviv (2019) 13(3):374–96. 10.1007/s11764-019-00759-9 PMC6548762 31055708

[33] Website of Hong Kong Cancer Registry. Hospital Authority (Accessed Accessed: December 2020). URL: www3.ha.org.hk/cancereg/statistics.html.

[34] Cheng FWT Lam GKS Cheuk DKL Luk CW Li CH Ling SC. on behalf of the Hong Kong Paediatric Haematology and Oncology Study Group. Report summarizes acute lymphoblastic leukemia study findings from Chinese University of Hong Kong (overview of treatment of childhood acute lymphoblastic leukaemia in Hong Kong). HK J Paediatr (New Series) (2019) 24:184–91.

[35] Conners CK Sitarenios G. Conners’ Continuous Performance Test (CPT). In: Kreutzer JS DeLuca J Caplan B, editors. Encyclopedia of Clinical Neuropsychology. New York, NY: Springer New York (2011). p. 681–3.

[36] Casarotti A Papagno C Zarino B. Modified Taylor Complex Figure: Normative data from 290 adults. J Neuropsychol (2014) 8(2):186–98. 10.1111/jnp.12019 23647550

[37] Strauss E Sherman EMS Spreen O. A compendium of neuropsychological tests. 3rd. New York: Oxford University Press (2006).

[38] Achenbach TM Rescorla LA. Manual for the ASEBA school-age forms & profiles: An integrated system of multi-informant assessment. In: Research Center for Children, Youth & Families. Burlington: University of Vermont (2001).

[39] Leung PW Kwong SL Tang CP Ho TP Hung SF Lee CC. Test-retest reliability and criterion validity of the Chinese version of CBCL, TRF, and YSR. J Child Psychol Psychiatry (2006) 47(9):970–3. 10.1111/j.1469-7610.2005.01570.x 16930392

[40] Wong MCS Jiang JY Tang J Lam A Fung H Mercer SW. Health services research in the public healthcare system in Hong Kong: An analysis of over 1 million antihypertensive prescriptions between 2004–2007 as an example of the potential and pitfalls of using routinely collected electronic patient data. BMC Health Serv Res (2008) 8(1):138. 10.1186/1472-6963-8-138 18578878 PMC2453117

[41] National Cancer Institute. Cancer Therapy Evaluation Program (CTEP). Common terminology criteria for adverse events (CTCAE). Available at: http://Ctep.cancer.gov/protocolDevelopment/electronic_applications/ctc.htm (Accessed December 2020).

[42] de Blank PMK Fisher MJ Lu L Leisenring WM Ness KK Sklar CA. Impact of vision loss among survivors of childhood central nervous system astroglial tumors. Cancer (2016) 122(5):730–9. 10.1002/cncr.29705 PMC4764436 26755438

[43] Shek DTL. Assessment of family functioning in Chinese adolescents: The Chinese family assessment instrument. Int Perspect Child Adolesc Ment Health (2002) 2(Supplement C):297–316. 10.1016/S1874-5911(02)80013-6

[44] Siu AM Shek DT. Psychometric properties of the Chinese family assessment instrument in Chinese adolescents in Hong Kong. Adolescence (2005) 40(160):817–30. 16468674

[45] Kong AP Choi KC Li AM Hui SS Chan MH Wing YK. Association between physical activity and cardiovascular risk in Chinese youth independent of age and pubertal stage. BMC Public Health (2010) 10:303–3. 10.1186/1471-2458-10-303 PMC2893096 20525239

[46] Chung OK Li HC Chiu SY Ho KY Lopez V. The impact of cancer and its treatment on physical activity levels and behavior in Hong Kong Chinese childhood cancer survivors. Cancer Nurs (2014) 37(3):43. 10.1097/NCC.0b013e3182980255 23842523

[47] Varni JW Limbers CA. The PedsQL™ Multidimensional Fatigue scale in young adults: Feasibility, reliability and validity in a university student population. Qual Life Res (2007) 17(1):105–14. 10.1007/s11136-007-9282-5 18027106

[48] Varni JW Burwinkle TM Katz ER Meeske K Dickinson P. The PedsQL in pediatric cancer: Reliability and validity of the pediatric quality of life inventory generic core scales, multidimensional fatigue scale, and cancer module. Cancer (2002) 94(7):2090–106. 10.1002/cncr.10428 11932914

[49] Ye Q Liu K Wang J Bu X Zhao L. Reliability and validity of the Chinese version of the PedsQL multidimensional fatigue scale in children with acute leukemia. Int J Nurs Sci (2016) 3(2):146–52. 10.1016/j.ijnss.2016.04.001

[50] Sun J Dunne MP Hou X Xu A. Educational stress scale for adolescents: development, validity, and reliability with Chinese students. J Psychoeducational Assessment (2011) 29(6):534–46. 10.1177/0734282910394976

[51] Benjamini Y Hochberg Y. Controlling the false discovery rate: A practical and powerful approach to multiple testing. J R Stat Society: Ser B (Methodological) (1995) 57(1):289–300. 10.1111/j.2517-6161.1995.tb02031.x

[52] Schultz KA Ness KK Whitton J Recklitis C Zebrack B Robison LL. Behavioral and social outcomes in adolescent survivors of childhood cancer: A Report from the Childhood Cancer Survivor Study. J Clin Oncol (2007) 25(24):3649–56. 10.1200/JCO.2006.09.2486 17704415

[53] Huang I Brinkman TM Kimberg CI Pui C Hudson MM Krull KR. Family environment, parent protection, and quality of life in childhood acute lymphoblastic leukemia (ALL) survivors. J Clin Oncol (2015) 33(15):e21027. 10.1200/jco.2015.33.15_suppl.e21027

[54] Cheung YT Brinkman TM Mulrooney DA Mzayek Y Liu W Banerjee P. Impact of sleep, fatigue, and systemic inflammation on neurocognitive and behavioral outcomes in long-term survivors of childhood acute lymphoblastic leukemia. Cancer (2017) 123(17):3410–9. 10.1002/cncr.30742 PMC5570612 28452142

[55] Hayes AF. Introduction to mediation, moderation, and conditional process analysis: A regression-based approach. 2nd ed. New York; London: Guilford Press (2018).

[56] Salzer WL Burke MJ Devidas M Dai Y Hardy KK Kairalla JA. Impact of intrathecal triple therapy versus intrathecal methotrexate on disease-free survival for high-risk B-lymphoblastic leukemia: Children’s oncology group study AALL1131. J Clin Oncol (2020) 38(23):2628–38. 10.1200/JCO.19.02892 PMC7402996 32496902

[57] Bhang S Yoon J Sung J Yoo C Sim C Lee C. Comparing attention and cognitive function in school children across noise conditions: A quasi-experimental study. Psychiatry Investig (2018) 15(6):620–7. 10.30773/pi.2018.01.15 PMC6018141 29940716

[58] Fowler PJ McGrath LM Henry DB Schoeny M Chavira D Taylor JJ. Housing mobility and cognitive development: Change in verbal and nonverbal abilities. Child Abuse Negl (2015) 48:104–18. 10.1016/j.chiabu.2015.06.002 PMC4593721 26184055

[59] Ferguson KT Cassells RC MacAllister JW Evans GW. The physical environment and child development: An international review. Int J Psychol (2013) 48(4):437–68. 10.1080/00207594.2013.804190 PMC4489931 23808797

[60] Owolabi O Zhang Z Wei X Yang N Li H Wong SYS. Patients’ socioeconomic status and their evaluations of primary care in Hong Kong. BMC Health Serv Res (2013) 13(1):487. 10.1186/1472-6963-13-487 24274660 PMC4222269

[61] Wong CSM Chan WC Lam LCW Law WY Tang WY Wong TY. Living environment and psychological distress in the general population of Hong Kong. Proc Environ Sci (2016) 36:78–81. 10.1016/j.proenv.2016.09.016

[62] Ni MY Yao XI Leung KSM Yau C Leung CMC Lun P. Depression and post-traumatic stress during major social unrest in Hong Kong: A 10-year prospective cohort study. Lancet (2020) 395(10220):273–84. 10.1016/S0140-6736(19)33160-5 31928765

[63] Tan J Tan J Yates S Yates S. Academic expectations as sources of stress in Asian students. Soc Psychol Educ (2011) 14(3):389–407. 10.1007/s11218-010-9146-7

[64] English A Zeng Z Ma J. The stress of studying in China: Primary and secondary coping interaction effects. SpringerPlus (2015) 4(1):1–14. 10.1186/s40064-015-1540-3 26693113 PMC4666850

[65] Hooke M Rodgers C Taylor O Koerner K Mitby P Moore I. Physical activity, the childhood cancer symptom Cluster–Leukemia, and cognitive function: A longitudinal mediation analysis. Cancer Nurs (2018) 41(6):434–40. 10.1097/NCC.0000000000000634 PMC6203650 30124481

[66] Huang IC Thompson LA Chi YY Knapp CA Revicki DA Seid M. The linkage between pediatric quality of life and health conditions: Establishing clinically meaningful cutoff scores for the PedsQL. Value Health (2009) 12(5):773–81. 10.1111/j.1524-4733.2008.00487.x PMC4299816 19508660

[67] Varni JW Burwinkle TM Seid M. The PedsQL™ as a pediatric patient-reported outcome: Reliability and validity of the PedsQL™ measurement model in 25,000 children. Expert Rev Pharmacoecon Outcomes Res (2014) 5(6):705–19. 10.1586/14737167.5.6.705 19807613

[68] Li WHC Ho KY Ho LLK Lam HS Lam KKW Chui SY. Adventure-based training to promote physical activity and reduce fatigue among childhood cancer survivors: A randomized controlled trial. Int J Nurs Stud (2018) 83:65–74. 10.1016/j.ijnurstu.2018.04.007 29689482

[69] Patsou ED Alexias GD Anagnostopoulos FG Karamouzis MV. Effects of physical activity on depressive symptoms during breast cancer survivorship: A meta-analysis of randomised control trials. ESMO Open (2017) 2(5):e000271. 10.1136/esmoopen-2017-000271 29259819 PMC5729305

[70] Garcia DO Thomson CA. Physical activity and cancer survivorship. Nutr Clin Pract (2014) 29(6):768–79. 10.1177/0884533614551969 PMC4470419 25335787

[71] Glisky E. Changes in Cognitive Function in Human Aging in: Brain Aging: Models, Methods, and Mechanisms Riddle DR. Boca Raton (FL): CRC Press/Taylor & Francis (2007). 21204355

[72] Cupit-Link MC Kirkland JL Ness KK Armstrong GT Tchkonia T LeBrasseur NK. Biology of premature ageing in survivors of cancer. ESMO Open (2017) 2(5):e000250. 10.1136/esmoopen-2017-000250 29326844 PMC5757468

[73] Carroll JE Van Dyk K Bower JE Scuric Z Petersen L Schiestl R. Cognitive performance in survivors of breast cancer and markers of biological aging. Cancer (2019) 125(2):298–306. 10.1002/cncr.31777 30474160 PMC7082843

[74] Zang Y. Impact of physical exercise on children with attention deficit hyperactivity disorders: Evidence through a meta-analysis. Med (Baltimore) (2019) 98(46):e17980. 10.1097/MD.0000000000017980 PMC6867774 31725664

[75] Cox CL Montgomery M Oeffinger KC Leisenring W Zeltzer L. Promoting physical activity in childhood cancer survivors: Results from the Childhood Cancer Survivor Study. Cancer (2009) 115(3):642–54. 10.1002/cncr.24043 PMC2653221 19117349

[76] Li HCW Chung OKJ Ho KY Chiu SY Lopez V. Effectiveness of an integrated adventure-based training and health education program in promoting regular physical activity among childhood cancer survivors. Psycho-Oncology (2013) 22(11):2601–10. 10.1002/pon.3326 23733273

[77] Fidler-Benaoudia MM Oeffinger KC Yasui Y Robison LL Winter DL Reulen RC. A comparison of late mortality among survivors of childhood cancer in the United States and United Kingdom. J Natl Cancer Inst (2020) djaa151. 10.1093/jnci/djaa151 33002115 PMC8096371

[78] Fidler MM Reulen RC Winter DL Kelly J Jenkinson HC Skinner R. Long term cause specific mortality among 34 489 five year survivors of childhood cancer in Great Britain: Population based cohort study. BMJ (2016) 354:i4351. 10.1136/bmj.i4351 27586237 PMC5008696

[79] Reulen RC Guha J Bright CJ Henson KE Feltbower RG Hall M. Risk of cerebrovascular disease among 13 457 five-year survivors of childhood cancer: A population-based cohort study. Int J Cancer (2021) 148(3):572–83. 10.1002/ijc.33218 32683688

[80] Hawkins MM Lancashire ER Winter DL Frobisher C Reulen RC Taylor AJ. The British Childhood Cancer Survivor Study: Objectives, methods, population structure, response rates and initial descriptive information. Pediatr Blood Cancer (2008) 50(5):1018–25. 10.1002/pbc.21335 17849473

[81] ElAlfy M Ragab I Azab I Amin S Abdel-Maguid M. Neurocognitive outcome and white matter anisotropy in childhood acute lymphoblastic leukemia survivors treated with different protocols. Pediatr Hematol Oncol (2014) 31(2):194–204. 10.3109/08880018.2013.871763 24498883

