# Supplementary material for: Neurocognitive and Behavioral Outcomes of Chinese Survivors of Childhood Lymphoblastic Leukemia
Source: Front Oncol. 2021 Apr 20;11:655669. doi: 10.3389/fonc.2021.655669 (PMC8093634; doi:10.3389/fonc.2021.655669)
Supplement: Supplementary file 1 [file DataSheet_1.docx]

Supplement 1: Neurocognitive and Behavioral Measures, and Reference Norms Data Sources

| Neurocognitive Outcomes | Measures and Domains | Reference Norms Data |
| --- | --- | --- |
| Attention | CPT-III ^1^  *Attentiveness:*  CPT detectability  CPT omissions  CPT variability  *Sustained attention:*  HRT block change  HRT ISI change | US norms ^1^ |
| Memory | Modified Taylor Complex Figure ^2^ | Age- and sex-matched norms (Casrotti et al. ) ^2^ |
| Motor processing speed | *Visual search:*  Trail Making A | Age-matched Chinese norms (Lee at al. ) ^6^ |
|  | *Motor processing speed:*  Grooved Pegboard ^3,4^ | Age- and sex-matched norms (Lafayette) ^5^ |
| Cognitive flexibility | *Cognitive flexibility:*  Trail Making Test B ^3^ | Age-matched Chinese norms (Lee at al. ) ^6^ |
|  | CPT commissions  CPT perseverations | US norms ^1^ |
|  |  |  |
| Behavioral Outcomes | **Measures and Domains** | **Reference Norms Data** |
|  | **Child Behavior Checklist/ Adult Behavior Checklist**  ^7^ | Multicultural norms (Hong Kong Chinese) provided by ASEBA |
| Syndrome Scales | Attention Problems (refer to features of inattention, hyperactivity, and impulsivity). |  |
|  | Thought Problems (refer to symptoms common in several mental disorders: hallucinations, OCD-symptoms, strange thoughts and behaviors, self-harm and suicide attempts) |  |
|  | Internalizing problems (refer to over-inhibited or internally-focused symptoms; includes subscales: *Anxious/Depressed; Withdrawn; Somatic Complaints*) |  |
|  | Externalizing problems (refer to disinhibited or externally-focused behavioral symptoms; included subscales *Aggressive Behavior; Rule-breaking Behavior, and Intrusive*) |  |
|  | Obsessive-Compulsive Problems (refer to obsessions and compulsions symptoms) |  |
|  | Sluggish Cognitive Tempo (refer to symptoms associated with inconsistent alertness and slowness in thinking) |  |

CPT-III: Conners Continuous Performance Test (3rd Edition)

1. Conners CK, Sitarenios G. Conners’ Continuous performance test (CPT). In: Kreutzer JS, DeLuca J, Caplan B, eds. *Encyclopedia of Clinical Neuropsychology.* New York, NY: Springer New York; 2011:681-683.
2. Casarotti A, Papagno C, Zarino B. Modified Taylor Complex Figure: Normative data from 290 adults. *Journal of Neuropsychology*. 2014;8(2):186-198
3. Strauss E, Sherman EMS, Spreen O. *A compendium of neuropsychological tests.* 3rd ed; 2006. Oxford University Press, Oxford, England
4. Merker B., Podell K. Grooved Pegboard Test. 2011. In: Kreutzer J.S., DeLuca J., Caplan B. (eds) Encyclopedia of Clinical Neuropsychology. Springer, New York
5. Grooved Pegboard Test User’s Manual. URL: <https://www.advys.be/docs/GroovedPegboardTestManual.pdf>. Accessed on June 1, 2020
6. Lee T, Yuen K, Chan CJ  Normative data for neuropsychological measures of fluency, attention, and memory measures for Hong Kong Chinese. Clin Exp Neuropsychol. 2002 Aug;24(5):615-32
7. Manual for the ASEBA school-age forms & profiles: An integrated system of Multi-informant assessment. 2001. Burlington: University of Vermont, Research Center for Children, Youth & Families

Supplement 2: Consort Flow Diagram

**Screened**

(n=192)

**Excluded (n=17)**

Relapsed (n=13)

Pre-existing cognitive impairment (n=3)

Secondary malignancy (n=1)

**Eligible**

(n=175)

Non-participants (n=17)

Contact not successful (default appointment [5], uncontactable [4]) (n=9)

Refusal (did not have time [5], not interested [1]) (n=6)

Withdrawal (did not have time to finish) (n=2)

Response rate: 86.9%

**Completed all assessments**

(n=158)

Incomplete diagnosis information [1] or treatment protocol [5] information (n=6)

**Analyzed**

(n=152)

Supplement 3: Clinical and Treatment Characteristics of Study Cohort Stratified by Cranial Radiation Therapy

| Characteristics | CRT group (n=32) | Non-CRT group (n=120) | *P* |
| --- | --- | --- | --- |
| Demographics and Clinical |  |  |  |
| Sex |  |  | **0.001** ^†^ |
| Male | 25 (78.1) | 54 (45.0) |  |
| Female | 7 (21.9) | 66 (55.0) |  |
| Highest education (years) *mean [SD]* | 14.2 [2.8] | 13.0 [3.4] | 0.054 ^Ɨ^ |
| Age at diagnosis (years) *mean [SD]* | 6.8 [4.6] | 6.1 [4.2] | 0.53 ^Ɨ^ |
| Age at evaluation (years) *mean [SD]* | 30.8 [6.8] | 21.5 [6.0] | **<0.0001** ^Ɨ^ |
| ≤ 18 | 1 (3.1) | 31 (25.9) |  |
| >18 – ≤30 | 14 (43.8) | 79 (65.8) |  |
| >30 | 17 (53.1) | 10 (8.3) |  |
| Time since diagnosis (years) *mean [SD]* | 24.0 [7.9] | 15.4 [6.4] | **<0.0001** ^Ɨ^ |
| 5 – ≤10 | 1 (3.1) | 26 (21.7) |  |
| >10 – ≤15 | 2 (6.3) | 33 (27.5) |  |
| >15 – ≤20 | 8 (25.0) | 36 (30.0) |  |
| >20 | 21 (65.6) | 25 (20.8) |  |
| Time since completion of treatment (years) *mean [SD]* | 20.9 [7.3] | 14.0 [11.5] | **<0.0001** ^Ɨ^ |
| 5 – ≤10 | 1 (3.1) | 38 (31.7) |  |
| >10 – ≤15 | 4 (12.5) | 31 (25.8) |  |
| >15 – ≤20 | 8 (25.0) | 35 (29.2) |  |
| >20 | 19 (59.4) | 16 (13.3) |  |
| Risk group |  |  | **<0.0001** ^†^ |
| Standard risk | 1 (3.1) | 56 (46.7) |  |
| Intermediate risk | 8 (25.0) | 57 (47.5) |  |
| High risk | 23 (71.8) | 5 (4.1) |  |
| *Missing* | 0 | 2 (1.6) |  |
| HSCT | 4 (12.5) | 0 | -- |
| Chemotherapy |  |  |  |
| IV daunorubicin/ doxorubicin* (mg/m^2^) *mean [SD]* | 220.6 (52.8) | 168.5 (53.2) | **<0.0001** |
| IV high-dose methotrexate* (mg/m^2^) *mean [SD]* | 10900 (9900) | 17500 (4600) | **0.024** |
| 8g/m^2^ | 6 (18.7) | 50 (41.6) |  |
| 20 g/m^2^ | 24 (81.3) | 70 (58.3) |  |
| Intrathecal chemotherapy* (no. of counts) *mean [SD]* | 16.4 (5.5) | 18.9 (5.4) | **<0.001** |
| Chronic health conditions^ |  |  |  |
| Any |  |  | **<0.0001** ^†^ |
| Cardiopulmonary | 5 (15.6) | 8 (6.7) | 0.10 ^†^ |
| Endocrine | 4 (12.5) | 3 (2.5) | **0.016** ^†^ |
| Metabolic | 4 (12.5) | 3 (2.5) | **0.016** ^†^ |
| Neurology | 6 (18.8) | 4 (3.3) | **0.002** ^†^ |
| Psychiatry | 2 (6.3) | 7 (5.8) | 0.92 ^†^ |
| Vision & Hearing | 0 | 3 (2.5) | -- |

HSCT: hematopoietic stem cell transplantation; IV: intravenous; SD: standard deviation

*Cumulative doses of selected chemotherapy drugs were extracted from medical charts, which were only available for 138 survivors. Cumulative doses for the remaining survivors (n=14) were estimated based on the chemotherapy protocol they received.

^ Conditions were graded for severity according to the National Cancer Institute’s Common Terminology Criteria for Adverse Events (CTCAE version 4.03).

^Ɨ^ Comparison between CRT group and non-CRT group was conducted using Mann-Whitney U test

^†^ Comparison between CRT group and non-CRT group was conducted using Chi-square test

Supplement 4: Socio-environmental Factors

| Variables |  | Total (n=152) | | CRT group (n=32) | | Non-CRT group (n=120) | |  |
| --- | --- | --- | --- | --- | --- | --- | --- | --- |
|  | Min-max | Mean (SD) | Range | Mean (SD) | Range | Mean (SD) | Range | *P* ^Ɨ^ |
| Physical activity# | 0-10 | 6.0 (1.6) | 1-10 | 6.0 (1.8) | 3-10 | 6.0 (1.5) | 1-10 | 0.79 |
| Academic stress^^,§^ | 16-80 | 52.5 (9.6) | 37-79 | 56.3 (15.5) | 41-79 | 52.2 (9.1) | 37-76 | 0.31 |
| Fatigue# | 0-100 | 68.3 (14.8) | 19.4-100 | 67.7 (18.4) | 19.4-95.8 | 68.4 (13.8) | 34.7-100 | 0.81 |
| General fatigue | 0-100 | 72.4 (18.6) | 20.8-100 | 70.1 (21.6) | 20.8-100 | 73.0 (17.8) | 29.2-100 |  |
| Sleep fatigue | 0-100 | 61.9 (17.3) | 20.8-100 | 63.2 (16.9) | 20.8-91.7 | 61.6 (17.5) | 20.8-100 |  |
| Cognitive fatigue | 0-100 | 70.5 (18.3) | 16.6-100 | 69.9 (22.1) | 16.7-100 | 70.6 (17.7) | 29.2-100 |  |
| Family functioning^ | 33-165 | 68.1 (21.4) | 33-130 | 69.1 (24.7) | 35-130 | 67.8 (20.5) | 33-172 | 0.75 |
| Communication | 9-45 | 21.8 (8.0) | 9-43 | 22.3 (9.1) | 10-43 | 21.7 (7.7) | 9-36 |  |
| Concern | 3-15 | 4.7 (2.0) | 3-13 | 4.8 (2.4) | 3-13 | 4.6 (1.9) | 3-10 |  |
| Conflict | 6-30 | 12.0 (4.1) | 6-25 | 12.1 (4.9) | 6-25 | 12.0 (3.9) | 6-24 |  |
| Control | 3-15 | 5.1 (2.0) | 3-11 | 5.4 (2.2) | 3-10 | 5.1 (2.0) | 3-11 |  |
| Mutuality | 12-60 | 24.5 (8.7) | 11-46 | 24.6 (9.3) | 12-46 | 24.4 (8.6) | 11-44 |  |
| Living space |  | n | % | n | % | n | % | *P* ^†^ |
| ≤ 600 square feet | -- | 90 | 59.2 | 17 | 53.1 | 73 | 60.8 | 0.43 |
| > 600 square feet | -- | 62 | 40.8 | 15 | 46.9 | 47 | 39.2 |  |

CRT: Cranial radiation therapy

^ A higher value was indicative of worse functioning

# A higher value was indicative of better functioning

^§^ Academic stress was evaluated in survivors who were still schooling

^Ɨ^ Comparison between CRT group and non-CRT group was conducted using general linear models, adjusting for sex, age at evaluation and age at diagnosis.

^†^ Comparison between CRT group and non-CRT group was conducted using logistic regression, adjusting for sex, age at evaluation and age at diagnosis

Supplement 5: Neurocognitive and Behavioral Outcomes (Overall Cohort)

|  |  | Neurocognitive outcomes | | | | | | |  |
| --- | --- | --- | --- | --- | --- | --- | --- | --- | --- |
|  | | | **Mean (SD)**  *T*-Scores* | **Median (IQR)**  *T*-Scores* | | **Impaired %**^ | **95% CI** | ***P***# | ***P*** ^Ɨ^ |
| Executive function | | |  |  |  |  |  |  |  |
| CPT Perseverations | | | 51.6 (7.3) | 54.5 | (52.0 – 55.0) | 4.0 | 1.4 – 7.5 | **0.009** | **0.011** |
| CPT Commission | | | 54.0 (7.8) | 56.0 | (51.0 – 59.0) | 8.5 | 4.1 – 12.9 | **0.027** | **0.031** |
| Cognitive flexibility (TMT-B) | | | 51.6 (23.1) | 43.5 | (39.1 – 53.7) | 5.3 | 2.0 – 9.2 | 0.49 | 0.50 |
| Attention | | |  |  |  |  |  |  |  |
| CPT HRT ISI change (sustained attention) | | | 50.4 (8.9) | 51.0 | (45.0 – 56.0) | 4.0 | 1.4 – 7.5 | 0.58 | 0.58 |
| CPT HRT block change (sustained attention) | | | 52.3 (9.0) | 52.0 | (48.0 – 56.7) | 8.5 | 4.1 – 12.9 | **0.003** | **0.004** |
| CPT Variability (attentiveness) | | | 51.8 (10.1) | 54.0 | (46.0 – 60.0) | 4.0 | 1.4 – 7.5 | **0.010** | **0.012** |
| CPT Omission (attentiveness) | | | 45.8 (4.6) | 45.0 | (43.0 – 46.0) | 0 | 0 | **<0.001** | **0.0014** |
| CPT Detectability (attentiveness) | | | 54.4 (9.8) | 56.0 | (48.3 – 61.0) | 10.5 | 5.4 – 15.6 | **<0.001** | **0.0014** |
| Memory | | | 57.0 (13.8) | 57.9 | (46.6 – 66.7) | 9.2 | 4.7 – 16.9 | **<0.001** | **0.0014** |
| Motor processing speed | | |  |  |  |  |  |  |  |
| Visual search (TMT-A) | | | 47.7 (13.7) | 46.0 | (45.0 – 49.0) | 4.6 | 1.8 – 8.3 | 0.43 | 0.47 |
| Motor processing speed (GPB) | | | 59.5 (12.7) | 57.6 | (51.2 – 65.8) | 36.2 | 29.3 – 44.2 | **<0.001** | **0.0014** |
|  |  | Behavioral outcomes (Syndrome scales) | | | | | | |  |
|  | | | **Mean (SD)**  *T*-Scores* | **Median** | **(IQR)** | **Impaired %**^ | **95% CI** | ***P***# |  |
| Attention problems | | | 56.1 (7.4) | 53.0 | (50.0 – 60.0) | 12.5 | 8.2 – 18.7 | **˂0.001** | **0.0014** |
| Thought problems | | | 56.3 (7.6) | 52.0 | (50.0 – 63.0) | 17.8 | 10.0 – 23.9 | **˂0.001** | **0.0014** |
| Internalizing problems | | | 54.3 (11.9) | 55.0 | (44.0 – 62.7) | 17.1 | 11.9 – 23.9 | **˂0.001** | **0.0014** |
| Anxious/Depressed | | | 56.6 (8.1) | 50.0 | (54.0 – 61.0) | 16.4 | 11.4 – 23.2 | **˂0.001** | **0.0014** |
| Somatic complaints | | | 57.3 (8.2) | 53.0 | (51.0 – 62.7) | 19.1 | 13.6 – 26.1 | **˂0.001** | **0.0014** |
| Withdrawn | | | 56.9 (8.1) | 53.0 | (51.0 – 61.0) | 15.8 | 10.8 – 22.4 | **˂0.001** | **0.0014** |
| Externalizing problems | | | 50.6 (10.7) | 50.5 | (43.2 – 57.0) | 7.9 | 4.6 – 13.3 | 0.46 | 0.49 |
| Aggressive behavior | | | 54.8 (6.3) | 52.0 | (50.0 – 58.0) | 7.9 | 4.6 –13.3 | **˂0.001** | **0.0014** |
| Intrusive | | | 53.5 (5.7) | 51.0 | (50.0 – 55.0) | 5.9 | 2.9 –11.6 | **˂0.001** | **0.0014** |
| Rule-breaking behavior | | | 54.4 (6.0) | 51.0 | (50.0 – 58.0) | 7.9 | 4.6 – 13.3 | **˂0.001** | **0.0014** |
| Obsessive-compulsive problems | | | 55.1 (7.1) | 51.0 | (50.0 – 58.0) | 9.9 | 6.1 – 15.6 | **˂0.001** | **0.0014** |
| Sluggish cognitive tempo | | | 57.5 (7.9) | 55.0 | (50.0 – 64.0) | 23.7 | 17.6 – 31.0 | **˂0.001** | **0.0014** |

AD/H: attention deficit/ hyperactivity; CI: confidence interval; CPT: Conners Continuous Performance Test-III; GPB: Grooved Pegboard; HRT: hit reaction time; ISI: inter-stimulus Intervals; SD: standard deviation; TMT: Trail Making Test

* All neurocognitive and behavioral measures were transformed into age-adjusted *T*-scores (mean = 50; standard deviation [SD] = 10) using references provided by the test manuals or the published literature (Supplement 1). All *T*-scores were scaled such that a higher score was indicative of worse functioning or more severe problems.

^ To estimate the prevalence of impairments within the study sample, impairment was defined as a score poorer than 1.5 standard deviation of age-adjusted *T*-scores of reference norms

# A one-sample t test was used to compare the survivors’ performance with population norms (*T*-score = 50). Boldface indicates statistical significance at *P*≦0.05.

^Ɨ^ Statistical significance after applying false discovery rate. Boldface indicates statistical significance at *P*≦0.05.

Supplement 6: Comparison of Neurocognitive and Behavioral Outcomes between Survivors Treated with and without Cranial Radiation Therapy

| Outcomes | CRT group (n=32) | | | Non-CRT group (n=120) | | |  |
| --- | --- | --- | --- | --- | --- | --- | --- |
| Neurocognitive outcomes | | | | | | | |
|  | Mean (SD)  *T*-Scores* | Impaired (%)^ | 95% CI (%) | Mean (SD)  *T*-Scores | Impaired (%)^ | 95% CI (%) | *P*# |
| Executive function |  |  |  |  |  |  |  |
| CPT Perseverations | 52.4 (7.3) | 3.1 | 1.4 – 9.9 | 51.3 (7.3) | 2.5 | 2.1 – 4.6 | 0.38 |
| CPT Commission | 55.2 (3.9) | 15.6 | 3.1 – 26.7 | 53.8 (8.5) | 6.6 | 2.3 – 14.2 | 0.53 |
| Cognitive flexibility (TMT-B) | 60.7 (28.2) | 18.8 | 6.7 – 36.6 | 48.9 (20.9) | 2.5 | 1.5 – 5.9 | **0.050** |
| Attention |  |  |  |  |  |  |  |
| CPT HRT ISI change (sustained attention) | 50.6 (9.9) | 3.1 | 1.4 – 9.9 | 50.3 (8.7) | 4.2 | 0.9 – 8.3 | 0.83 |
| CPT HRT block change (sustained attention) | 50.1 (7.6) | 3.1 | 1.4 – 9.9 | 52.8 (9.4) | 10.0 | 5.3 – 16.4 | 0.57 |
| CPT Variability (attentiveness) | 52.2 (10.8) | 6.2 | 1.6 – 9.7 | 51.7 (10.0) | 3.3 | 2.8 – 3.9 | 0.72 |
| CPT Omission (attentiveness) | 46.2 (8.3) | 0 |  | 45.7 (3.1) | 0 |  | 0.97 |
| CPT Detectability (attentiveness) | 54.7 (11.7) | 15.6 | 3.1 – 26.7 | 54.3 (9.3) | 9.4 | 4.3 – 15.2 | 0.95 |
| Memory | 54.4 (16.0) | 20.0 | 8.1– 41.6 | 57.8 (13.2) | 6.6 | 2.3 – 14.2 | 0.63 |
| Motor processing speed |  |  |  |  |  |  |  |
| Visual search (TMT-A) | 46.5 (12.5) | 6.2 | 1.6 – 9.7 | 48.2 (13.2) | 4.2 | 0.9 – 8.3 | 0.63 |
| Motor processing speed (GPB) | 54.8 (9.9) | 31.2 | 13.3 – 45.5 | 60.7 (13.1) | 37.5 | 30.2 – 46.8 | 0.081 |
| Behavioral outcomes (Syndrome scales) | | | | | | | |
|  | Mean (SD)  *T*-Scores* | Impaired %^ | 95% CI | Mean (SD)  *T*-Scores | Impaired %^ | 95% CI | *P*# |
| Attention problems | 56.0 (9.7) | 12.5 | 5.0 – 28.1 | 56.1 (6.8) | 12.5 | 7.7 – 19.6 | 0.34 |
| Thought problems | 57.3 (10.0) | 25.0 | 13.3 – 42.1 | 56.0 (6.8) | 15.8 | 10.4 – 23.4 | 0.26 |
| Internalizing problems | 54.1 (16.2) | 31.3 | 18.0 – 48.6 | 54.3 (10.6) | 13.3 | 8.4 – 20.6 | 0.97 |
| Anxious/Depressed | 58.3 (11.1) | 25.0 | 13.3 – 42.1 | 56.1 (7.1) | 14.2 | 9.0 – 21.5 | 0.18 |
| Somatic complaints | 57.5 (9.0) | 28.1 | 15.6 – 45.4 | 57.3 (8.1) | 16.7 | 11.1 – 24.3 | 0.50 |
| Withdrawn | 58.1 (10.8) | 21.9 | 11.0 – 38.8 | 56.6 (7.2) | 14.2 | 9.0 – 21.5 | 0.34 |
| Externalizing problems | 49.8 (13.8) | 12.5 | 5.0 – 28.1 | 50.8 (9.8) | 6.7 | 3.4 – 12.6 | 0.62 |
| Aggressive behavior | 55.9 (8.2) | 12.5 | 5.0 – 28.1 | 54.5 (5.6) | 6.7 | 3.4 – 12.6 | 0.60 |
| Intrusive | 53.4 (6.0) | 9.7 | 3.3 – 24.9 | 53.6 (5.6) | 4.5 | 1.8 – 11.1 | 0.96 |
| Rule-breaking behavior | 53.9 (7.0) | 9.4 | 3.2 – 24.2 | 54.6 (5.7) | 7.5 | 4.0 – 13.6 | 0.64 |
| Obsessive-compulsive problems | 57.1 (8.8) | 18.8 | 8.9 – 35.3 | 54.6 (6.5) | 7.5 | 4.0 – 13.6 | **0.048** |
| Sluggish cognitive tempo | 57.4 (8.8) | 28.1 | 15.6 – 45.4 | 57.5 (7.7) | 22.5 | 15.9 – 30.8 | 0.97 |

AD/H: attention deficit/ hyperactivity; CI: confidence interval; CRT: cranial radiation therapy; CPT: Conners Continuous Performance Test-III; GPB: Grooved Pegboard; HRT: hit reaction time; ISI: inter-stimulus Intervals; SD: standard deviation; TMT: Trail Making Test

* All neurocognitive and behavioral measures were transformed into age-adjusted *T*-scores (mean = 50; standard deviation [SD] = 10) using references provided by the test manuals or the published literature (Supplement 1). All *T*-scores were scaled such that a higher score was indicative of worse functioning or more severe problems.

^ To estimate the prevalence of impairments within the study sample, impairment was defined as a score poorer than 1.5 standard deviation of age-adjusted *T*-scores of reference norms

# Multiple linear regression was used to compare *T*-scores between CRT versus non-CRT group. All statistical models were adjusted for sex, age at evaluation and age at diagnosis. Boldface indicates statistical significance at *P*≦0.05.

Supplement 8: Inter-correlation among Socio-environmental Factors

| Factors | Physical activity# | | Fatigue# | | Academic stress^ | | Family functioning^ | |
| --- | --- | --- | --- | --- | --- | --- | --- | --- |
|  | r* | *P* | r* | *P* | r* | *P* | r* | *P* |
| Physical activity# | 1.00 | -- | -- | -- | -- | -- | -- | -- |
| Fatigue# | 0.34 | **<0.0001** | 1.00 | -- | -- | -- | -- | -- |
| Academic stress^^,§^ | 0.097 | 0.38 | -0.40 | **<0.0001** | 1.00 | -- | -- | -- |
| Family functioning^ | -0.11 | 0.15 | -0.33 | **<0.0001** | 0.38 | **<0.0001** | 1.00 | -- |

* Spearman’s correlation test was conducted to examine the bivariate correlation among socio-environmental factors. Boldface indicates statistical significance at *P*≦0.05.

^ A higher value was indicative of worse functioning

# A higher value was indicative of better functioning

^§^ Academic stress was evaluated in survivors who were still schooling

Supplement 9: Exploratory Mediation Analysis

| **Behavioral Outcomes** | **Total Effect** | | **Indirect Effect** | | | | | | |
| --- | --- | --- | --- | --- | --- | --- | --- | --- | --- |
|  |  |  | **Total Indirect Effect** | | | **Fatigue** | | **Physical Activity** | |
|  | ß | *P* | ß | 95% BCCI | *P* | ß | *P* | ß | *P* |
| Attention problems | 5.55 | **0.001** | 3.12 | 1.27-5.81 | **0.006** | 3.06 | **0.007** | 0.06 | 0.77 |
| Thought problems | 3.02 | 0.072 | 3.02 | 1.26-5.66 | **0.005** | 3.07 | **0.007** | -0.05 | 0.79 |
| Internalizing problems | 6.03 | **0.016** | 5.60 | 2.44-9.34 | **0.001** | 6.03 | **0.001** | -0.43 | 0.30 |
| Externalizing problems | 4.68 | **0.032** | 3.92 | 1.55-7.37 | **0.007** | 4.24 | **0.005** | -0.32 | 0.41 |
| Sluggish cognitive tempo | 5.32 | **0.003** | 3.69 | 1.56-6.01 | **0.001** | 3.71 | **0.001** | -0.02 | 0.91 |

ß: Standardized estimates refer to standardized coefficient estimates for each path. The standardized coefficients reflect the relative strengths among the paths, such that the effects of different paths can be evaluated on the same scale

BCCI: Bias corrected confidence intervals for mediation analysis in which fatigue and physical activity were represented as mediators in the association between chronic health conditions and behavioral outcomes (controlling for sex, age at evaluation, age at diagnosis and cranial radiation)

Supplement 10: Comparison between Participants and Non-participants

| Characteristics | Participants  (n = 152) | Non-participants  (n = 23) | *P* |
| --- | --- | --- | --- |
| Sex *n (%)* |  |  |  |
| Male | 79 (52.0) | 15 (65.2) | 0.12 |
| Female | 73 (48.0) | 8 (34.8) |  |
| Age at diagnosis (years) *mean [SD]* | 6.3 (4.3) | 7.8 (4.3) | 0.09 |
| Age at evaluation (years) *mean [SD]* | 23.5 (7.2) | 25.2 (7.9) | **0.03** |
| Time since diagnosis (years) *mean [SD]* | 17.2 (7.6) | 17.6 (7.3) | 0.80 |

Comprehensive clinical and treatment data in non-participants are not available, as extracting patient information from electronic health records for research purposes is strictly prohibited if the patient declined participation.
